# Elucidating the Regulatory Elements for Transcription Termination and Posttranscriptional Processing in the Streptomyces clavuligerus Genome

**DOI:** 10.1128/mSystems.01013-20

**Published:** 2021-05-04

**Authors:** Soonkyu Hwang, Namil Lee, Donghui Choe, Yongjae Lee, Woori Kim, Yujin Jeong, Suhyung Cho, Bernhard O. Palsson, Byung-Kwan Cho

**Affiliations:** a Department of Biological Sciences, Korea Advanced Institute of Science and Technology, Daejeon, South Korea; b KAIST Institute for the BioCentury, Korea Advanced Institute of Science and Technology, Daejeon, South Korea; c Department of Bioengineering, University of California San Diego, La Jolla, California, USA; d Department of Pediatrics, University of California San Diego, La Jolla, California, USA; e Novo Nordisk Foundation Center for Biosustainability, Technical University of Denmark, Lyngby, Denmark; f Intelligent Synthetic Biology Center, Daejeon, South Korea; g Innovative Biomaterials Research Center, KAIST Institutes, Korea Advanced Institute of Science and Technology, Daejeon, South Korea; FMRP-USP

**Keywords:** *Streptomyces*, transcription termination, term-seq, transcription unit, 3′ untranslated region

## Abstract

Identification of transcriptional regulatory elements in the GC-rich *Streptomyces* genome is essential for the production of novel biochemicals from secondary metabolite biosynthetic gene clusters (smBGCs). Despite many efforts to understand the regulation of transcription initiation in smBGCs, information on the regulation of transcription termination and posttranscriptional processing remains scarce. In this study, we identified the transcriptional regulatory elements in β-lactam antibiotic-producing Streptomyces clavuligerus ATCC 27064 by determining a total of 1,427 transcript 3′-end positions (TEPs) using the term-seq method. Termination of transcription was governed by three classes of TEPs, of which each displayed unique sequence features. The data integration with transcription start sites and transcriptome data generated 1,648 transcription units (TUs) and 610 transcription unit clusters (TUCs). TU architecture showed that the transcript abundance in TU isoforms of a TUC was potentially affected by the sequence context of their TEPs, suggesting that the regulatory elements of TEPs could control the transcription level in additional layers. We also identified TU features of a xenobiotic response element (XRE) family regulator and DUF397 domain-containing protein, particularly showing the abundance of bidirectional TEPs. Finally, we found that 189 noncoding TUs contained potential *cis*- and *trans*-regulatory elements that played a major role in regulating the 5′ and 3′ UTR. These findings highlight the role of transcriptional regulatory elements in transcription termination and posttranscriptional processing in *Streptomyces* sp.

**IMPORTANCE** *Streptomyces* sp. is a great source of bioactive secondary metabolites, including antibiotics, antifungal agents, antiparasitic agents, immunosuppressant compounds, and other drugs. Secondary metabolites are synthesized via multistep conversions of the precursor molecules from primary metabolism, governed by multicomplex enzymes from secondary metabolite biosynthetic gene clusters. As their production is closely related with the growth phase and dynamic cellular status in response to various intra- and extracellular signals, complex regulatory systems tightly control the gene expressions related to secondary metabolism. In this study, we determined genome-wide transcript 3′-end positions and transcription units in the β-lactam antibiotic producer Streptomyces clavuligerus ATCC 27064 to elucidate the transcriptional regulatory elements in transcription termination and posttranscriptional processing by integration of multiomics data. These unique features, such as transcript 3′-end sequence, potential riboregulators, and potential 3′-untranslated region (UTR) *cis*-regulatory elements, can be potentially used to design engineering tools that can regulate the transcript abundance of genes for enhancing secondary metabolite production.

## INTRODUCTION

*Streptomyces* spp. are Gram-positive bacteria, with GC-rich genomes. They have been the focus of scientific interest due to their ability to produce a vast range of secondary metabolites, including antibiotics, antifungal agents, antiparasitic agents, immunosuppressant compounds, and other drugs (1–3). These secondary metabolites are usually produced by well-coordinated biosynthetic reactions involving complex multienzymes encoded in secondary metabolite biosynthetic gene clusters (smBGCs) (4). Each *Streptomyces* genome encodes more than 30 smBGCs, and their products are diverse, both chemically and biologically (5). Despite their diversity, only a small portion of these secondary metabolites have been produced in laboratory settings due to the silent expression of smBGCs (6). The expression of these genes is tightly regulated by complex transcriptional regulatory networks in response to dynamic environmental signals (7, 8).

Several transcriptional regulatory elements, including sigma factors, pleiotropic transcriptional regulators, and pathway-specific transcriptional regulators, have been reported in *Streptomyces* strains (4, 7, 9, 10). Recent studies have also found that bacterial genomes include regulatory elements not only for transcription initiation but also for transcription termination and posttranscriptional processing (11, 12). Several high-throughput techniques have been applied to a broad range of bacterial species to identify these regulatory elements. For example, genome-scale transcription 3′-end termini information has uncovered various regulatory traits in bacterial genomes (13), such as Rho-independent and -dependent terminators (14, 15), antibiotic-responsive riboregulators (16), evolutionary convergence of stoichiometry between genes in the same operon (17), discordant transcript abundance resulting from RNase cleavage activity (18, 19), and pervasive bidirectional transcription terminators (20).

However, these regulatory elements controlling transcription termination and posttranscriptional processing in *Streptomyces* spp. have not been studied in detail compared with other bacteria. It was expected that *Actinobacteria* may have unique regulatory elements of transcription termination for smBGC regulations because the genes of smBGCs are located close to each other on the genome; thus, transcription termination is expected to be tightly regulated to avoid transcriptional interference (21). Moreover, the transcript abundance of each smBGC gene is also expected to be rapidly regulated at the posttranscriptional level in response to dynamic environmental changes (22). Several distinct features of transcription termination and posttranscriptional processing in *Actinobacteria* relative to those of Escherichia coli have been reported. For example, in transcription termination, key residues involved in Rho’s oligomerization, ATP hydrolysis, RNA binding, and RNA translocation are highly conserved through bacterial genomes, but *Actinobacteria* often contain additional *rho* duplicates and/or Rho proteins bearing the insertion domains of unknown functions (23). One of Rho duplicates of Streptomyces lividans bears mutations in the bicyclomycin binding domain and RNA binding domain that may contribute to poor sensitivity to bicyclomycin and different RNA binding modes, respectively (23). In addition, posttranscriptional processing in *Actinobacteria* regulates secondary metabolism and morphological differentiation (22). Particularly, RNase III positively controls actinorhodin and prodiginine production in Streptomyces coelicolor (24), jadomycin production in Streptomyces venezuelae (25), and antinomycin production in Streptomyces antibioticus (26). RNase J affects jadomycin production in *S. venezuelae* (25), and oligoribonuclease is required for aerial mycelium in *S. griseus* (27). Although the regulation targets and mechanisms of these nucleases have not been fully elucidated, they were considered to be related to mRNA stability and rRNA processing affecting translation (22). Recently, genome-scale determination of transcript 3′-end positions (TEPs) in S. lividans revealed their unique features, and a potential transcript stability regulation site in ectoine BGC was suggested as an example (28). However, this is far from a great enough number when considering there are more than 1,000 reported *Streptomyces* strains.

Here, we determined the genome-wide transcript 3′-end positions (TEPs), transcription units (TUs), and transcription unit clusters (TUCs) through the integration of term-seq data and other multiomics data (29), in order to elucidate the major transcriptional regulatory elements controlling transcription termination and posttranscriptional processing in Streptomyces clavuligerus ATCC 27064. *S. clavuligerus* is an industrial strain used for the production of clavulanic acid, a β-lactamase inhibitor, and contains 58 smBGCs in its genome—among the highest number across *Streptomyces* species—including a 1.8-Mb megaplasmid (29, 30). We also analyzed continuous RNA transcripts between the 5′ and 3′ end, which included processed mRNAs and noncoding RNAs, for the elucidation of regulatory elements for both transcription termination and posttranscriptional processing.

## RESULTS

### Term-seq determines genome-wide TEPs.

**FIG 1:**
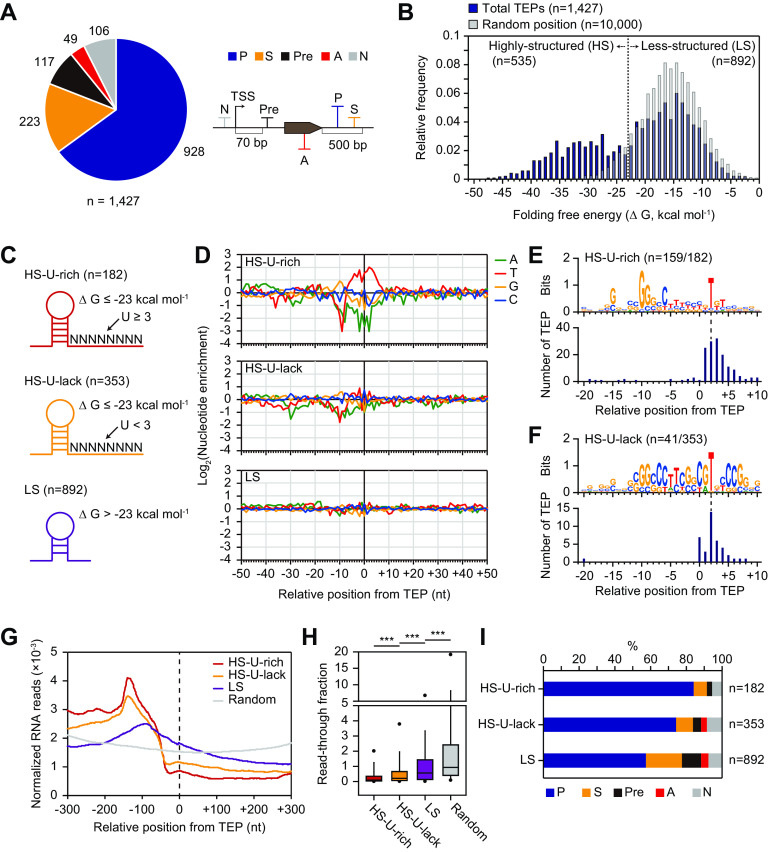
Unique features of transcript 3′-end positions (TEPs) in Streptomyces clavuligerus. (A) Categorization of 1,427 TEPs based on their relative position from the gene. P, primary; S, secondary; Pre, premature; A, antisense; and N, intergenic. (B) Folding free energy distribution of the predicted RNA structures of the 0- to 40-bp upstream sequence from the total TEPs (*n* = 1,427) and random positions (*n* = 10,000). The TEPs were divided into highly structured (HS) and less structured (LS) TEPs with folding free energy values lower than and higher than −23 kcal/mol, which is the middle value (−23 kcal/mol) of the bimodal distribution, respectively. (C) Schematic representation of the three TEP classes depending on the folding free energy of their stem structure and the uridine number in the 8-bp tract downstream of the stem. (D) Nucleotide enrichment around the three TEP classes (C). (E and F) Enriched sequence motifs at the −20 to +10 position were associated with HS-U-rich TEPs (E) and HS-U-lack TEPs (F). (G) Normalized RNA read density from positions −300 bp to +300 bp were associated with the three classes of TEPs and random positions. (H) Distribution of the read-through fraction, which is the average normalized RNA read-count ratio of positions −300 bp to 0 bp to positions 0 bp to +300 bp downstream from the 3 classes of TEP and random positions at the 4 growth phases. Outliers of ≥5% were discarded. ***, *P < *0.001 (Mann-Whitney *U* test, two-sided). (I) Proportion of TEPs in each category of A for the three TEP classes of C.

TEPs in the *S. clavuligerus* genome were identified using term-seq with high quality and reproducibility (see Fig. S1, Fig. S2A and B, and Data Set S1, sheet 1 in the supplemental material) (16). A total of 1,769 TEPs were determined by selecting the enriched mapped peaks using machine learning and manual curation. Among them, 342 internal TEPs located inside the open reading frame (ORF) were excluded from this study, as we focused on TEPs in the intergenic region. Consequently, 1,427 TEPs were identified (Fig. S2C) and grouped into 5 categories based on their genomic positions relative to adjacent genes, as follows (Fig. 1A): (i) 928 TEPs were categorized as primary TEPs (P), which showed the highest peak intensity among the TEPs located at the 500-bp downstream region of the associated gene; (ii) 223 TEPs in the same region, except that primary TEPs were categorized as secondary TEPs (S); (iii) 117 TEPs were categorized as premature TEPs (Pre) and were located at the region between 70 bp downstream of the primary TSS and the first position of the start codon for the associated gene for ensuring proper transcription termination; (iv) 49 TEPs located within the ORF of the opposite strand were categorized as antisense TEPs (A); and (v) the remaining 106 TEPs were categorized as intergenic TEPs (N).

10.1128/mSystems.01013-20.1 FIG S1 A schematic illustration of term-seq for determination of genome-scale TEPs. (A) Growth profile of *S. clavuligerus* in R5− medium (B) The sequencing library construction protocol for term-seq. (C) An overview of processing and mapping of the sequencing reads. Download FIG S1, TIF file, 0.3 MB. Copyright © 2021 Hwang et al. 2021 Hwang et al. https://creativecommons.org/licenses/by/4.0/ This content is distributed under the terms of the Creative Commons Attribution 4.0 International license.

10.1128/mSystems.01013-20.2 FIG S2 Validation of term-seq quality. (A) Statistics of term-seq. Rep1 and Rep2 were duplicate samples from extracted RNAs of a previous study. (B) Correlation of reads per million (RPM) accumulated at each gene (first base of the start codon to 500-bp downstream position from 3′ end of the stop codon) between two term-seq replicates. Coefficient of determination (R^2^) was indicated. (C) An example of a term-seq profile mapped onto the *S. clavuligerus* genome. Normalized log_2_ RNA-seq read count at four growth phases (E, early; T, transition; L, late exponential; S, stationary) were represented with the term-seq 3′-end read count. Red vertical lines with a short horizontal line were determined TEPs by specific criteria, which are described in the Materials and Methods. Download FIG S2, TIF file, 0.8 MB. Copyright © 2021 Hwang et al. 2021 Hwang et al. https://creativecommons.org/licenses/by/4.0/ This content is distributed under the terms of the Creative Commons Attribution 4.0 International license.

10.1128/mSystems.01013-20.10 DATA SET S1 Sheet 1. List of all transcript 3′ end sites (TEPs), transcription units (TUs), and transcription unit clusters (TUCs). For TEP category: P, primary; S, secondary; Pre, premature; A, antisense; N, intergenic. Folding free energy of the RNA structure is calculated from the sequence at −40 bp upstream to 0 bp from TEP (kcal/mol). Avg. readthrough fraction is readthrough fraction, which is the average normalized RNA read count ratio of −300 bp upstream to the 0-bp position and 0-bp to +300-bp downstream position from the TEP. For TU category: Mono, monocistronic TU; Poly, polycistronic TU; Pre, premature TU; Inter, intergenic RNA. RPF_cut is the criteria for a potential small protein for which the RPKM of RPF median average value is bigger than the median value of CDSs (18.75), and average of log_2_ RPKM ratio of RPF/RNA is bigger than the log_2_ median ratio of CDSs (0.74). ORF frame is marked as “o” when the potential ORF having both start codon and stop codon was predicted within the TU. TSS_Cat and TEP_Cat are the categories of TSS and TEP, respectively. Nc_TU_Cat is the category of the noncoding intergenic TU. Information indicated the predicted noncoding RNA from the Rfam database or potential binding region of the gene. Sheet 2. The information of total 24 XRE-DUF397 gene pairs and all bidirectional TEP pairs with their upstream gene functions. Bi-TEP is the bidirectional TEP of XRE-DUF397 TUCs. TUCs having Bi-TEP are marked as “o.” Data Set S1, XLSX file, 0.3 MB Copyright © 2021 Hwang et al. 2021 Hwang et al. https://creativecommons.org/licenses/by/4.0/ This content is distributed under the terms of the Creative Commons Attribution 4.0 International license.

### Genome-wide analysis of TEPs shows unique transcriptional regulatory elements.

The identified TEPs were classified as transcription termination sites (TTSs) based on intrinsic termination or Rho-dependent termination, but some also included 3′-end positions enriched for posttranscriptional processing by RNase (14). Intrinsic terminators formed an RNA stem-loop structure, followed by a U-rich tract (31). Recent *in vivo* mapping of the 3′ end of Rho-dependent transcripts have revealed that they are enriched for an upstream stable RNA stem-loop structure that is protected from 3′ to 5′ exonuclease digestion (14). Thus, the RNA stem-loop structure is one of the most crucial regulatory elements for transcription termination and degradation at the 3′ end (32). As the *S. clavuligerus* genome has a high GC ratio (72.5%), the sequence context of the RNA stem-loop at the TEPs was expected to be different from those of other well-studied bacteria, such as Escherichia coli and Bacillus subtilis (14, 15).

The folding free energy (ΔG) of the RNA secondary structures for the 40-bp upstream sequence from TEPs (*n* = 1,427) and random genomic positions (*n* = 10,000) was calculated at 30°C (Fig. 1B). The absolute value of folding free energy for primary TEPs (P) was significantly higher than that for the other categories of TEPs, indicating that a high absolute value of folding free energy at the TEP is directly related to transcription termination (see Fig. S3A in the supplemental material). The folding free energy distribution in TEPs was bimodal, with median peaks at −32 kcal/mol and −15 kcal/mol, respectively. The bimodal distribution of the TEPs led us to divide them into two classes based on the middle folding free energy value (−23 kcal/mol) in which the two distributions intersect, and included highly structured (HS, *n* = 535) and less structured (LS, *n* = 892) TEPs.

10.1128/mSystems.01013-20.3 FIG S3 Characteristics of three different TEPs. (A) Folding free energy of predicted RNA structures that are 0- to 40-bp upstream sequences from 5 TEP categories described in Fig. 1A. Statistical significances were indicated for *P* value (***, *P < *0.001; **, *P < *0.01; ns, *P > *0.05; Mann-Whitney U test). (B) Distribution of the uridine number at 8 bp downstream RNA from the end of the stem of highly structured (HS) and less-structured (LS) TEP. (C to E) TEP stem information of three different TEP classes described in Fig. 1C. The distribution of the numbers of TEPs was analyzed for stem length (C), loop length (D), and stem-end position (E). (F) Alignment of RNA sequences from the −40- to +20-bp position relative to different groups of TEPs described in Fig. 1C. (G) Interaction frequency between two nucleotides located at the 100-bp upstream region of TEPs. HS-U-rich (left, upper triangle), HS-U-lack (middle, upper triangle), and LS (right, upper triangle) TEP groups were compared with random positions (lower triangle of all three squares). (H) Log_2_ scale RNA expression level of the genes that have the three different TEP classes described in Fig. 1C at the four growth phases. Statistical significances between HS-U-rich and HS-U-lack TEPs for each growth phase are indicated (*, *P < *0.05; ns, *P > *0.05; Mann-Whitney U test) above the graphs of HS-U-lack TEPs. Also, statistical significances between HS-U-lack and LS TEPs for each growth phase are indicated (*, *P < *0.05; ns, for *P > *0.05; Mann-Whitney U test) above the graphs of LS TEPs. Download FIG S3, TIF file, 2.1 MB. Copyright © 2021 Hwang et al. 2021 Hwang et al. https://creativecommons.org/licenses/by/4.0/ This content is distributed under the terms of the Creative Commons Attribution 4.0 International license.

HS-TEPs were expected to include intrinsic terminators that usually have a U-rich tract regulating RNA polymerase release together with the stem-loop structure (33). Therefore, HS-TEPs were additionally divided into two classes based on their uridine counts for 8 bp downstream of the RNA stem-loop structure; HS-U-rich TEPs (*n* = 182) were defined as three or more uridines, and HS-U-lack TEPs (*n* = 353) were defined as less than three uridines (Fig. 1C). The uridine count criteria (U = 3) was decided based on the frequency of TEPs with the largest difference between HS-TEPs and LS-TEPs (Fig. S3B). HS-U-rich and HS-U-lack TEPs showed a longer stem length and shorter loop length distribution than LS-TEPs, while the 3′-end position of the stem relative to the TEP was unchanged between the groups (Fig. S3C, D, and E). Previous reports of intrinsic terminators in E. coli supported these results that longer stem length increased the stability of the stem-loop and the termination efficiency (34). Also, the optimal loop length of intrinsic terminators in E. coli was 4 to 8 bp, similar to HS-U-rich and HS-U-lack TEPs, thereby supporting the fact that shorter or longer loops sterically inhibit the stability of the stem-loop resulting in decreased termination efficiency (20, 34, 35).

Next, nucleotide enrichment at the −50- to +50-nucleotide (nt) position relative to the TEP was evaluated to identify any distinct features between the three TEP classes (Fig. 1D). HS-U-rich TEPs contained (i) GC-rich stem-loops at the −30 to −10 region, (ii) U-rich tracts at the −5 to +5 region, and (iii) A-tracts at the −50 to −40 region. Because these features were identical to those of previously determined E. coli intrinsic terminators (14), they were considered intrinsic terminators in *S. clavuligerus*. However, there were some differences in the positions of the A-tracts and GC stem-loops between the two species. The A-tract was located at the −30 to −25 region in E. coli, which is about +20 nt downstream of those in *S. clavuligerus*. Also, the GC stem-loops were located at the −20 to −10 region in E. coli, which is about +10 nt downstream of those in *S. clavuligerus*. Additionally, the enrichment of cytosine in the −30 region and guanine in the −10 region was unique to *S. clavuligerus*. In contrast, the HS-U-lack TEPs lacked U-rich tracts and A-tracts but contained GC stem-loops at lower levels than the HS-U-rich TEPs. These sequence features and high absolute folding free energy values were comparable to those previously observed for the I-shaped terminator, which is highly conserved in GC-rich eubacterial genomes (36), as well as the recently identified 3′-terminal pattern of Rho-dependent termination sequences in E. coli (14). The LS-TEPs showed no nucleotide enrichment other than low enrichment of A-rich tracts at the −50 to −20 region. Presumably, they were either Rho-dependent termination sites with diffused termination patterns (33) or other distinct types of termination or RNA processing sites. Conserved motif search and sequence alignment of the −40 to +20 region confirmed the distinct characteristics of the three TEP classes; most HS-U-rich TEPs (87%) showed conserved GC stem-loops and U-rich tracts, while only a small portion of HS-U-lack TEPs (12%) showed conserved U-rich tracts at the +2 nt position; no enriched sequences were observed for LS-TEPs (Fig. 1E and F and Fig. S3F). Although LS-TEPs showed no distinct sequence characteristics, the interaction frequency between each base at 100 bp upstream of the TEPs suggested that LS-TEPs had weak RNA structures (Fig. S3G).

These distinct sequence features were expected to include transcriptional regulatory elements that could possibly decrease transcript abundance, and indeed, RNA abundance was dramatically decreased at the −100 to −1 region for all three TEP classes (Fig. 1G). For a quantitative representation of this decrease in transcript abundance, the transcription read-through fraction was calculated, which is the normalized RNA read count ratio of the +300-bp to –300-bp region relative to the TEP. Significantly lower read-through fractions were observed in HS-U-rich TEPs, followed by HS-U-lack, LS, and random (Mann-Whitney *U* test; ***, *P < *0.001; two-sided) (Fig. 1H). These results confirmed that HS-U-rich TEPs were the most efficient at decreasing transcript abundance among the three classes. Moreover, the transcript levels of the genes with primary (P) or secondary (S) HS-U-rich TEPs were higher than those of genes with the other TEP classes (Fig. S3H). In contrast, LS-TEPs showed a higher proportion of secondary (S), premature (Pre), and antisense (A) TEPs, which are possibly related to functionally different transcriptional regulatory mechanisms, such as condition-specific termination and posttranscriptional processing (Fig. 1I). Collectively, 1,427 determined TEPs showed unique regulatory features that may affect transcriptional termination and posttranscriptional processing for different TEP classes based on folding free energy, nucleotide enrichment, and changes in transcription levels.

### TSS and TEP integration determine genome-wide TU and TUC architecture.

**FIG 2:**
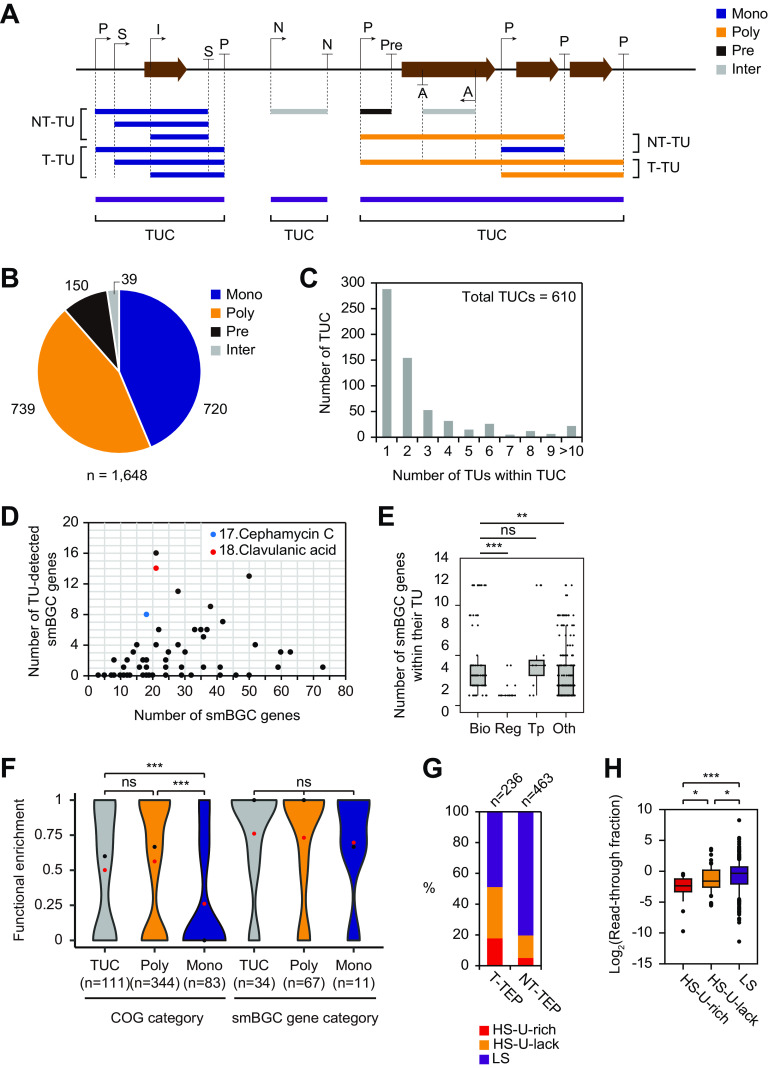
Characterization of transcription units (TUs) and transcription unit clusters (TUCs). (A) A schematic illustration of TUs and TUCs. Black arrows and black vertical lines with a short horizontal line indicate TSSs and TEPs with their categories, respectively. Terminal TUs (T-TU) and nonterminal TUs (NT-TU) are denoted by colors based on their TU categories, including monocistronic (Mono), polycistronic (Poly), premature (Pre), and intergenic (Inter). (B) Statistics of the 1,648 TUs. (C) Distribution of the number of TUs within each TUC. (D) The number of TU-assigned genes within 58 secondary metabolite biosynthetic gene clusters (smBGCs). (E) The number distribution of genes within their TUs for four different smBGC functional categories, including biosynthetic (Bio), regulatory (Reg), transport (Tp), and others (Oth). Each point in the graph indicates the number of smBGC genes within the TU of each gene. Statistical significance was indicated for *P* value (***, *P < *0.001; **, *P < *0.01; not significant [ns], *P > *0.05; Mann-Whitney *U* test, two-sided). (F) Clusters of Orthologous Groups and smBGC functional enrichment between the genes within the TUC, within polycistronic TU (Poly), and a monocistronic TU (Mono) gene with its adjacent genes. Red dots and black dots indicate the average and median, respectively. Statistical significance was determined based on the *P* value (***, *P < *0.001; ns, *P > *0.05; Mann-Whitney *U* test, two-sided). (G) Proportion of the three TEP classes of terminal TEP (T-TEP) and nonterminal TEP (NT-TEP). (H) Distribution of log_2_ average read-through fractions of the three classes of NT-TEPs at the four growth phases. Statistical significance is indicated using *P* values (***, *P < *0.001; *, *P < *0.05; Mann-Whitney *U* test, two-sided).

To determine the TU architecture, TSS information and transcriptome sequencing (RNA-seq) data were integrated with TEP information (Fig. 2A and Data Set S1, sheet 1) (29). TU was defined as the connected region between a TSS and a TEP, where the average RNA read count of every 200-bp window moved by 1 bp within the region was higher than 5% of the average RNA read count of the entire region. In addition to TUs that were generally single coding RNA transcripts from the TSS to the TTS, all continuous RNA transcripts included processed mRNAs and noncoding RNAs. A total of 1,648 TUs were evenly distributed across the genome; they were categorized based on the number of genes in each TU and their location relative to the genes (Fig. 2B). A total of 720 TUs with 1 gene were categorized as monocistronic (Mono), and 739 TUs having more than 1 gene were categorized as polycistronic (Poly). Of the remaining noncoding TUs with no genes, 150 ending with a premature TEP were categorized as premature (Pre) and 39 were categorized as intergenic (Inter). Some TUs were overlapped with each other, indicating that they may be the TU isoforms of the longest connected TUs. We defined a group of these TU isoforms as a transcription unit cluster (TUC) (Fig. 2A) (37). A total of 610 TUCs were determined, of which 47% (*n* = 289) involved only 1 TU, while the rest (*n* = 321) involved more than 1 TU (Fig. 2C and Data Set S1, sheet 1). The distribution of the gene numbers within each TUC showed a similar pattern with the TU numbers, except 77 TUCs with no gene (see Fig. S4A in the supplemental material).

10.1128/mSystems.01013-20.4 FIG S4 Transcription unit architecture in *S. clavuligerus*. (A) Distribution of the number of genes within each TUC. (B) Functional categorization of the total 1,554 smBGC genes predicted by antiSMASH. “Biosynthetic” and “biosynthetic-additional” categories from antiSMASH prediction were integrated to the biosynthetic category, and an additional “other” category which was known as “biosynthetic genes” from previous studies was added to the biosynthetic category. (C) Distribution of the number of TUs within each smBGC. smBGCs having more than one TU were represented as their smBGC number and the name. Download FIG S4, TIF file, 0.6 MB. Copyright © 2021 Hwang et al. 2021 Hwang et al. https://creativecommons.org/licenses/by/4.0/ This content is distributed under the terms of the Creative Commons Attribution 4.0 International license.

**FIG 3:**
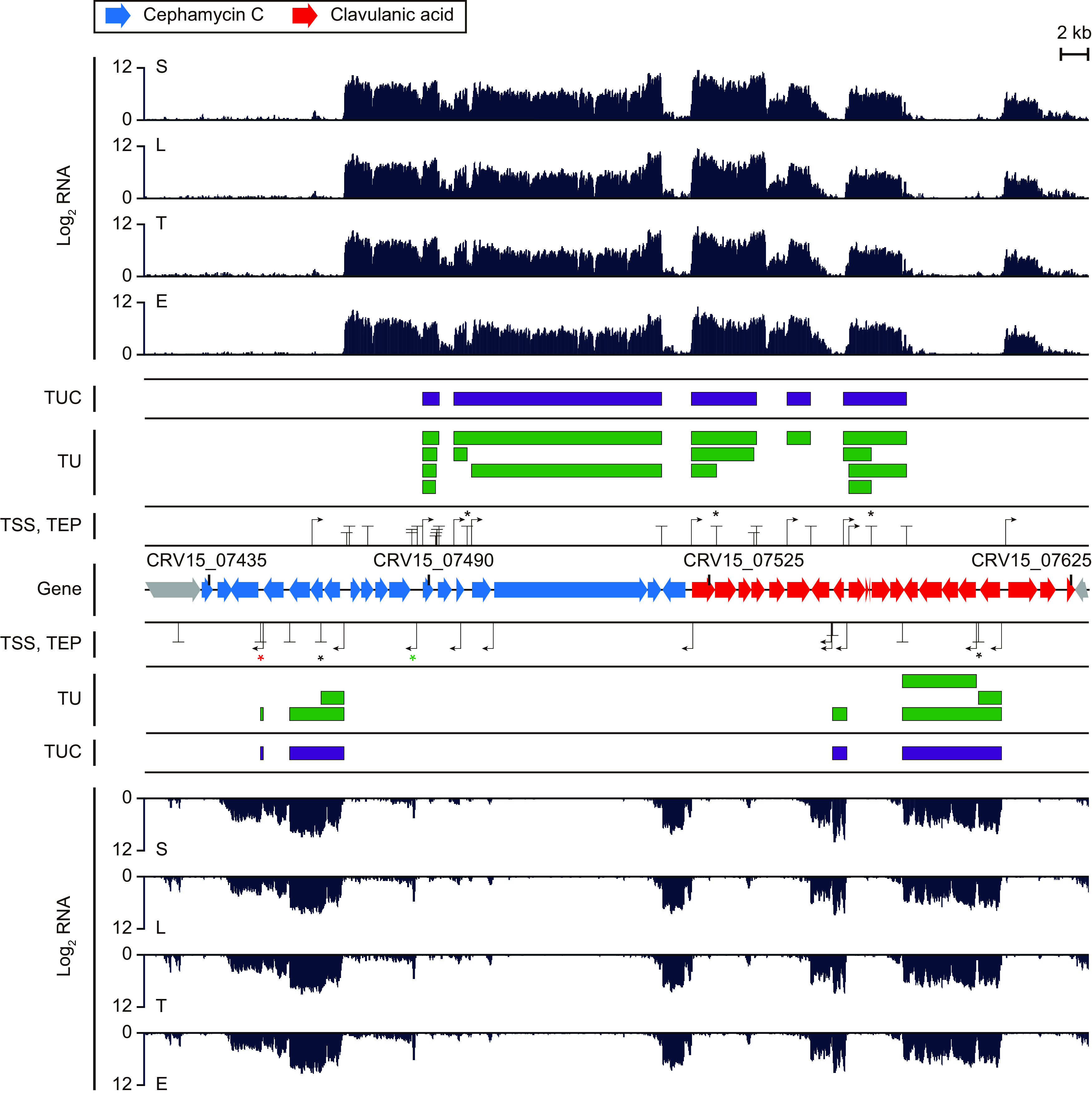
Transcription units (TUs), transcription unit clusters (TUCs), and expression profile of cephamycin C and clavulanic acid clusters at four different growth phases. Blue genes, cephamycin C cluster genes; red genes, clavulanic acid cluster genes; black arrow, TSS; black vertical line with a short horizontal line, TEP; green boxes, TU; purple boxes, TUC. The five black asterisks (*) indicate nonterminal TEPs. The red asterisk indicates the premature TU of CRV15_07445, and the green asterisk indicates the TSS of potential 3′-UTR *cis*-regulatory RNA at the *cmcH*-*ccaR* intergenic region.

For 58 smBGCs in the *S. clavuligerus* genome, a total of 160 TUs were found; of the total 1,554 smBGC genes, 163 were assigned to more than 1 TU (Fig. S4B and C). The two representative smBGCs for the biosynthesis of cephamycin C and clavulanic acid had 9 TUs for 8 genes and 12 TUs for 14 genes, respectively (Fig. 2D and Fig. 3). To examine how these TUs were clustered according to gene function, the 1,554 identified genes were divided into 4 different types according to their antiSMASH functional category (38)—“biosynthetic,” “regulatory,” “transport,” and “other” (Fig. S4B). The number of genes within the smBGC TUs was significantly higher for biosynthetic genes than for regulatory or other genes (Fig. 2E). This is because the biosynthetic genes in smBGCs were mainly involved in serial biosynthetic reactions in the same pathway; therefore, the coregulation of their transcription by a polycistronic TU would be a more efficient use of limited RNA polymerases and cellular resources (18). In contrast, the transcription of regulatory genes in smBGCs was usually regulated in response to dynamic environmental signals at the upper level of the regulatory network, in contrast to the biosynthetic genes; therefore, their regulation by monocistronic TUs would be more efficient (7).

### The TUC is a functional unit regulated by nonterminal TEPs.

TUCs with more than one gene included the longest polycistronic TUs, where the transcription of genes was coregulated for efficient regulation. Thus, we hypothesized that the gene functions within the TUCs and the polycistronic TUs were related. To examine this hypothesis, we calculated the ratio of the number of genes falling into one Clusters of Orthologous Groups (COG) category to the number of total COG categories for the genes. The COG functional enrichment of genes within the TUCs and polycistronic TUs was significantly higher than that of the monocistronic TUs and adjacent genes (Fig. 2F).

Although TUCs and polycistronic TUs are efficient for the coregulation of functionally related genes, they may be less useful when individual genes require differential expression, depending on the conditions. In this case, individual genes may be expressed through the activity of TU isoforms. TU isoforms in a TUC can be differentially expressed depending on the conditions at the transcription initiation step via the differential binding of transcription initiation factors to the specific sequence context around each alternative TSS (39). Likewise, TU isoforms in a TUC are also expected to be differentially expressed depending on the conditions at the transcription termination and posttranscriptional processing steps, owing to the differential regulation of termination or processing factors based on the specific sequence context of each alternative TEP (18).

To find the correlation between different sequence contexts for alternative TEPs and the transcript abundance of TU isoforms, we categorized TUs and TEPs within TUC according to their relative positions (Fig. 2A). Nonterminal TUs (NT-TUs) are TUs having their 3′-end position in the middle of TUC, which are named as nonterminal TEPs (NT-TEPs). Terminal TUs (T-TUs) are TUs having their 3′-end position at the 3′-end of TUC, which are named as terminal TEPs (T-TEPs). NT-TEPs included a higher proportion of LS-TEPs and a lower proportion of HS-TEPs than T-TEPs (Fig. 2G). In addition, the read-through fraction of the three classes of NT-TEPs had significantly higher values for LS-TEPs, followed by HS-U-lack and HS-U-rich TEPs (Fig. 2H). These results suggested that most NT-TEPs were LS-TEPs that might be condition-specific Rho-dependent termination sites or posttranscriptional processing sites (37). Moreover, the differential read-through fraction according to the sequence context of NT-TEPs suggested that NT-TEPs might be regulatory elements controlling transcript abundance between genes within the same TUC. For example, five out of nine TUCs within the smBGCs of cephamycin C and clavulanic acid contained NT-TUs involving different combinations of genes (Fig. 3; see Fig. S5 in the supplemental material). Compared with the reverse transcriptase PCR (RT-PCR) results of previous studies (40, 41), we identified the same TUs previously detected, as well as newly detected TUs (Fig. S5). NT-TEPs were located at the 3′-end intergenic region of *cefD*, *blp*, *ceaS2*, *fd*, and *pbpA* (Fig. 3). Two of them (*cefD* and *fd*) were LS-TEPs, while the other three (*blp*, *ceaS2*, and *pbpA*) were HS-U-rich TEPs. The read-through fractions of the two LS-TEPs were 1.63 and 1.2, while those of the three HS-U-rich TEPs were 0.06, 0.42, and 0.72 (Data Set S1, sheet 1). These NT-TEPs confirmed the relationship between transcript abundance changes and the sequence context of NT-TEPs. In particular, *ceaS2* governs the unique first condensation step of clavulanic acid biosynthesis, which may be needed to be regulated separately from downstream biosynthetic genes by HS-U-rich NT-TEPs to induce large changes in transcript abundance (Fig. 3 and Fig. S5) (42). Collectively, we can suggest that the TUC is a broad functional unit and that the transcript abundance of individual genes within the TUC may be regulated via the differential expression level of TU isoforms according to the sequence context of NT-TEPs.

10.1128/mSystems.01013-20.5 FIG S5 Transcription unit of cephamycin C and clavulanic acids cluster genes. (A) Overall biosynthetic pathway of cephamycin C. (B) Overall biosynthetic pathway of clavulanic acid. (C) Transcription unit architecture of cephamycin C cluster. (D) Transcription unit architecture of clavulanic acid cluster. Transcription units from this study and previous studies (40, 41) were represented as green and grey boxes, respectively. Blue genes were biosynthetic, orange genes were related to antibiotic resistance, green genes were transporter, red genes were regulatory proteins, and grey genes were unknown. For visual simplicity, only TU isoforms formed by primary TEPs were shown, while TU isoforms formed by secondary TEPs were not shown. Download FIG S5, TIF file, 0.9 MB. Copyright © 2021 Hwang et al. 2021 Hwang et al. https://creativecommons.org/licenses/by/4.0/ This content is distributed under the terms of the Creative Commons Attribution 4.0 International license.

### XRE-DUF397 is the most abundant transcriptional regulator related to secondary metabolism.

**FIG 4:**
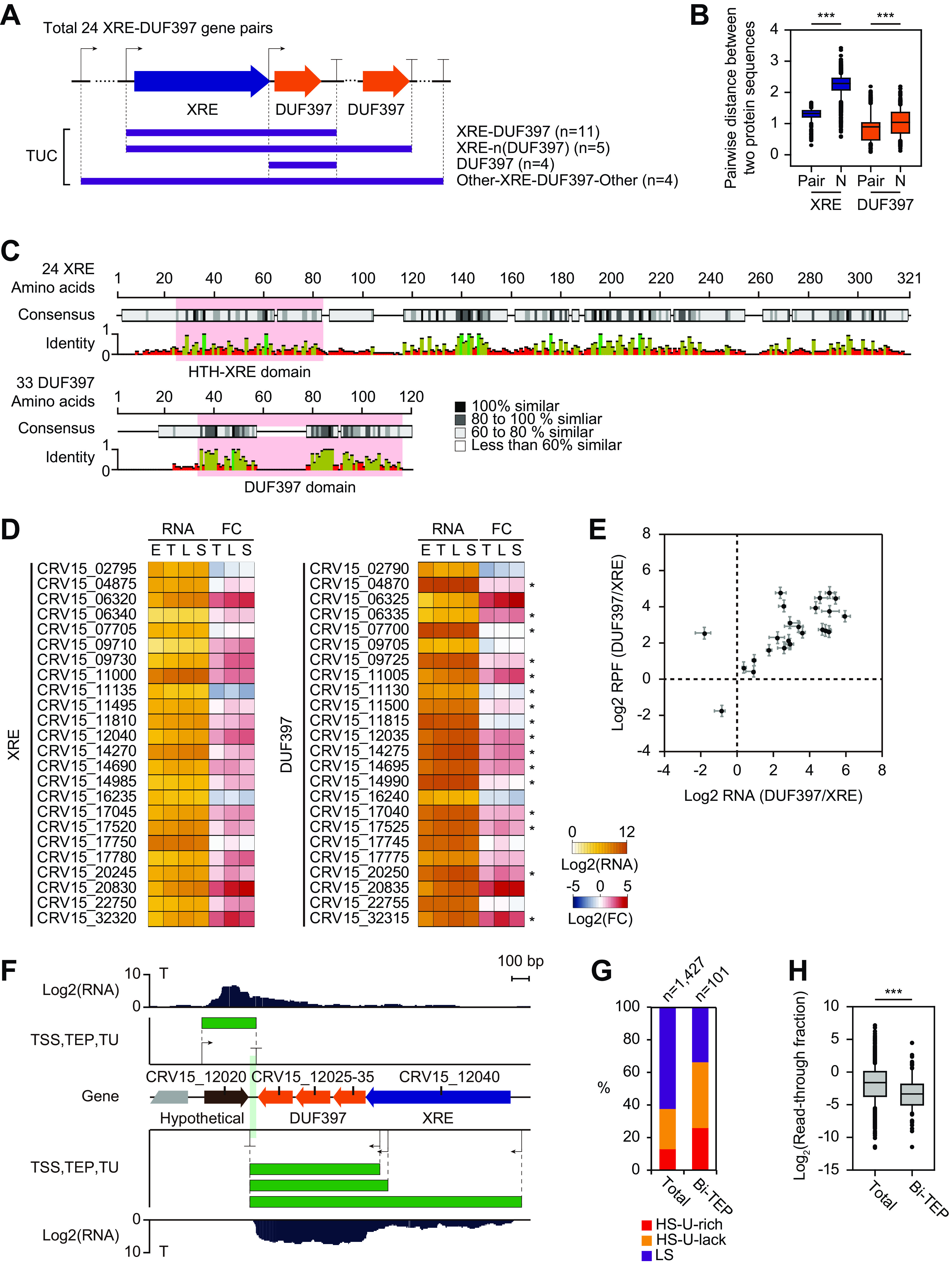
Transcription unit architecture of an XRE family regulator protein (XRE) and DUF397-domain-containing protein (DUF397) gene pair. (A) Transcription unit cluster (TUC) types of 24 XRE-DUF397 pairs in the Streptomyces clavuligerus genome. (B) Distribution of pairwise distance between paired and unpaired XRE protein sequences, and the distance between paired and unpaired DUF397 protein sequences. Pairwise distances were estimated using the Poisson model through ClustalW alignment with the BLOSUM protein weight matrix. Statistical significance is indicated using *P* values (***, *P < *0.001; Mann-Whitney *U* test, two-sided). (C) ClustalW alignment of 24 XRE and 33 DUF397 paired proteins. Sequence similarity and identity are indicated by the color and peak height, respectively. Conserved domains determined using the Pfam database (65) are highlighted using red boxes. (D) Expression levels and fold changes of 24 XRE-DUF397 gene pairs at the 4 growth phases. Fold change was determined at three growth phases, including transition (T), late-exponential (L), and stationary (S) phases, in comparison with the early-exponential phase (E). DUF397 genes indicated with asterisks (*) had alternative TSSs in addition to the TSSs of cognate XRE genes. (E) Correlation between the RNA and RPF ratio of DUF397 to XRE at the four growth phases; standard deviation is indicated using crossed lines. (F) An example of XRE-(n)DUF397 TU with bidirectional TEPs. The transparent vertical green box indicates overlapping regions of the bidirectional TEPs. (G) Proportion of the three TEP classes of total TEPs (total) and bidirectional TEPs (Bi-TEP). (H) Distribution of log_2_ average read-through fraction of total TEPs (total) and bidirectional TEPs (Bi-TEP) at the four growth phases. Statistical significance was indicated using *P* values (***, *P < *0.001; *, *P < *0.05; Mann-Whitney *U* test, two-sided).

One of the most notable findings of our genome-wide characterization of TUs and TUCs was the abundance of TUCs from the XRE family transcriptional regulator-DUF397 domain-containing protein/gene pair (Data Set S1, sheet 2). XRE is the most abundant transcriptional regulator with pleiotropic functions in the *S. clavuligerus* genome (7). XRE-DUF397 pairs are known to be homologous to the type II toxin (DUF397)-antitoxin (XRE) system, although DUF397 is not toxic to the host (43). A total of 74 XRE genes have been identified, comprising 20% of all transcriptional regulators (see Fig. S6A in the supplemental material) (7). Among them, 24 XRE genes were found to be adjacent to 33 downstream DUF397 genes (XRE-DUF397 gene pair) whose functions are currently unknown. Surprisingly, 20 of the 24 XRE-DUF397 gene pairs were included in TUCs, namely, XRE-DUF397 type (11 TUCs), XRE-(n)DUF397 type with tandem DUF397s (5 TUCs), and Other-XRE-DUF397-Other type (4 TUCs). The remaining TUCs were single-DUF397 types (Fig. 4A). Moreover, XRE-DUF397 gene pairs were also abundant in other *Streptomyces* genomes (Fig. S6B). According to a comparative genomics study of Streptomyces pratensis strains, XRE-DUF397 pairs were paralogous, as they were duplicated and coevolved through the simultaneous rapid accumulation of mutations (44). Sequence alignment of the paired XREs and paired DUF397s showed many single-nucleotide variations, and a strong positive correlation between the pairwise distances of two XREs and the pairwise distances of two DUF397s for two XRE-DUF397 pairs was observed (Fig. 4B and C; see Fig. S7 in the supplemental material).

10.1128/mSystems.01013-20.6 FIG S6 Abundance of XRE-DUF397 pairs in *Streptomyces* sp. (A) The number of regulator genes of the *S. clavuligerus* genome located within TUC and not located within TUC. Top 12 abundant regulatory functions were represented, and others were indicated as “others.” Among XRE genes, 24 XRE followed by DUF397 genes were indicated as “XRE-DUF397,” and 50 other XRE genes were indicated as “XRE-Other.” (B) Proportion of the XRE-DUF397 and XRE-other genes among total genes of 21 *Streptomyces* species. Download FIG S6, TIF file, 0.8 MB. Copyright © 2021 Hwang et al. 2021 Hwang et al. https://creativecommons.org/licenses/by/4.0/ This content is distributed under the terms of the Creative Commons Attribution 4.0 International license.

10.1128/mSystems.01013-20.7 FIG S7 Full ClustalW alignment of 24 XRE and 33 DUF397 paired proteins. The amino acid sequence of each protein and the consensus sequence were indicated. Sequence identity was indicated as the peak height. Black arrows with the alphabet-number were the conserved residues of DUF397 proteins from previous studies. Download FIG S7, TIF file, 1.9 MB. Copyright © 2021 Hwang et al. 2021 Hwang et al. https://creativecommons.org/licenses/by/4.0/ This content is distributed under the terms of the Creative Commons Attribution 4.0 International license.

The pairwise distance of amino acid sequences between paired XREs or DUF397 was significantly lower than that between other combinations of XRE or DUF397. This finding suggests that XRE-DUF397 pairs have distinct biological roles compared with unpaired XRE or DUF397 (Fig. 4B). For example, the WhiJ (XRE)-SCO4542 (DUF397) pair, in which SCO4542 represses WhiJ, and the unpaired BldB (DUF397) in S. coelicolor play important roles in broad cellular functions, including morphogenesis (aerial hyphae formation), antibiotic production, and catabolite control (45–47). It was recently shown that the Scr1-Scr2 pair of S. coelicolor acts as a strong positive regulator of multiple antibiotic production in multiple *Streptomyces* species (48). These results show that XRE-DUF397 pairs may be pleiotropic regulators as well as activators of secondary metabolism.

The RNA expression patterns of the XRE-DUF397 pairs were also diverse, but most showed an increase during later growth phases, consistent with the expression patterns of most smBGC genes (Fig. 4D). A majority of XRE-DUF397 pairs showed a much higher expression of DUF397 than of XRE, and alternative TSSs were observed upstream of most DUF397 sequences. In addition, five TUCs contained tandem DUF397 genes corresponding to one XRE. The interaction of one XRE with DUF397 multimers was expected, which is consistent with the results of a previous multimerization domain study of BldB (46). Indeed, the RNA and the ribosome-protected mRNA fragment (RPF) ratio of DUF397 to XRE within each XRE-DUF397 pair was consistently >1 and had a broad range (Fig. 4E).

Last, 10 of the 24 XRE-DUF397 pairs had bidirectional TEPs (Bi-TEPs) that overlapped at regions shorter than 60 bp on the opposite strand, and the ratio was unusually high compared with that for the 55 total Bi-TEPs observed across the genome (Fig. 4F and Data Set S1, sheet 2). Bi-TEPs are known to cause efficient transcription termination on both strands through transcriptional interference by RNA polymerase collision, which pauses transcription at the RNA stem structure (20). Here, the Bi-TEPs showed a significantly higher absolute value of folding free energy, longer stem length, and lower read-through fraction than those for total TEPs (Fig. 4G and H; see Fig. S8A in the supplemental material). Additionally, the loop length of Bi-TEPs was shorter than that of total TEPs, and the 3′-end position of the stem showed no difference (Fig. S8B and C). These results suggest that a high folding free energy and long stem length may elevate the pausing efficiency of RNA polymerase at the Bi-TEP. Bi-TEPs are also expected to stimulate efficient termination for the simultaneous coregulation of genes encoded on both strands (20). Most of the 10 XRE-DUF397 pairs with Bi-TEPs had an antisense gene encoding a hypothetical protein, although the functional correlation between them is unknown (Data Set S1, sheet 2). However, some of these genes included aminoglycoside phosphotransferase, endonuclease, and HAD family phosphatase. Moreover, four of the eight *mutT* pseudogenes, which have NUDIX hydrolase function, were located downstream of the XRE-DUF397 pairs on the same strand (49). All of these genes have common “house-cleansing” features to remove excess toxins or toxic intermediates. We can therefore suggest that *S. clavuligerus* has many intermediates based on the large number of smBGCs that require resistance genes, and each XRE-DUF397 pair may play a role in a specific secondary metabolic function and is tightly regulated by Bi-TEPs, with the antisense genes displaying resistance or cleansing functions.

10.1128/mSystems.01013-20.8 FIG S8 TEP stem information of total TEP (*n* = 1,427) and bidirectional TEP (*n* = 101). (A to C) The number frequency distribution of stem length (A), loop length (B), and stem-end position (C) were analyzed. Download FIG S8, TIF file, 0.5 MB. Copyright © 2021 Hwang et al. 2021 Hwang et al. https://creativecommons.org/licenses/by/4.0/ This content is distributed under the terms of the Creative Commons Attribution 4.0 International license.

### Identification of noncoding TUs and prediction of their potential targets.

**FIG 5:**
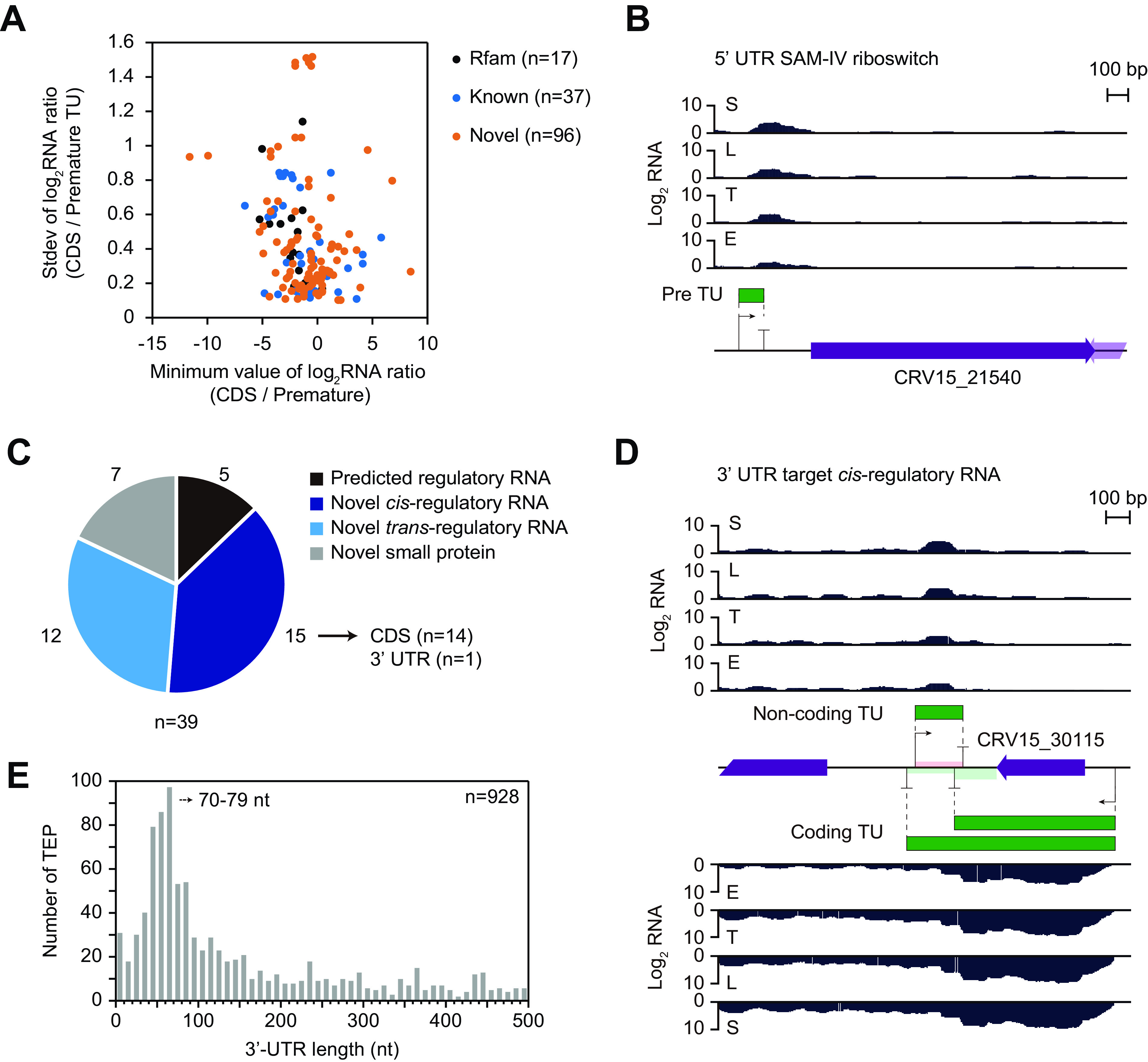
Identification of novel premature transcription units and noncoding RNAs. (A) Distribution of the minimum value of log_2_ RNA read-count ratio of the CDSs and premature TUs at the 4 growth phases, and standard deviation of the log_2_ RNA read-count ratio for 150 premature TUs. Premature TUs were categorized as predicted TUs using the Rfam database (Rfam), known TUs from previous studies (known), and previously unknown TUs (novel). (B) An example of the premature TU of CRV15_21540, which was predicted as SAM-IV riboswitch. (C) Classification of 39 intergenic TUs predicted as noncoding RNAs as per the Rfam database; novel *cis*-regulatory RNAs located at the antisense strand of the coding TUs; novel *trans*-regulatory RNAs not located at the antisense strand of the coding TUs, but expected to target distinct loci; and novel potential small protein TUs with sufficient RPF levels. (D) An example of novel *cis*-regulatory RNA expected to regulate antisense coding TU by binding at its 3′ UTR. (E) The length distribution of 3′ UTRs in *S. clavuligerus*. The most abundant length (70 to 79 nt) is indicated. Note that the result is calculated using primary TEPs but not using secondary TEPs.

Genome-wide determination of the continuous RNA transcripts involved the identification of noncoding TUs. A total of 150 premature TUs were determined with premature TEPs at the 3′-end termini (Data Set S1, sheet 1). A total of 17 premature TUs were riboswitches, as predicted by the Rfam database (50), and 37 premature TUs were identified in previous studies, including the attenuation by a ribosome protein leader (51–54). The remaining 96 premature Tus were novel. All three categories of premature TUs showed comparable distributions in terms of the minimum value of RNA read count ratio of premature TU to downstream coding DNA sequences (CDSs) among four growth phases (Fig. 5A). One of the most extreme decreases in the minimum value of the RNA read count ratio was observed for CRV15_21540, which encodes an aminotransferase class V-fold pyridoxal phosphate (PLP)-dependent enzyme; its premature TU was predicted to have a SAM-IV riboswitch (Fig. 5B) (55).

Likewise, other premature TUs were also expected to involve novel regulatory elements at the 5′ UTR. A total of 39 noncoding intergenic TUs were found (Fig. 5C and Data Set S1, sheet 1), of which 5 regulatory RNAs were predicted from the Rfam database (50) and the others were novel. Among these novel ones, some intergenic TUs with high RPF profiles were predicted to encode small proteins that were not detected by our annotation criteria. Therefore, seven intergenic TUs were categorized as potential small proteins according to the following criteria: (i) a higher average RPF value than the median RPF value of the total CDS (18.75), (ii) a higher average RPF/RNA ratio than the median RPF/RNA ratio of the total CDS (0.74), and (iii) potential ORF including start codon and stop codon within the TU. These small proteins were expected to encode short peptides composed of less than 100 amino acids involved in cellular processes such as transcriptional regulation, protein cleavage, stress response, and membrane proteins (56).

Among the remaining noncoding TUs, 15 were located within the antisense strand of the CDS containing a *cis*-regulatory element, and another 12 were located in the intergenic region with *trans*-regulatory elements for distant RNAs or other regulatory roles (11). One *cis*-regulatory RNA was located on the antisense strand of the 3′ UTR of CRV15_30115, which may regulate the RNA stability or transcription termination of the gene (Fig. 5D) (12). This *cis*-regulatory TU included a Bi-TEP, indicating that the expression of both the *cis*-regulatory TU and CRV15_30115 may be fine-tuned for efficient termination (Data Set S1, sheet 2).

The long 3′-UTR length distribution of *S. clavuligerus* calculated from primary TEPs suggests that 3′-UTR regulation by the binding of *cis*-regulatory RNAs and regulatory RNA-binding proteins and 3′-UTR derived *trans*-regulatory sRNAs may be more prevalent in *Streptomyces* sp. (Fig. 5E). Moreover, 181 genes were found to have alternative TEPs at their 3′ UTR (Fig. S9A). For example, the intergenic TSS was located within the antisense strand of the alternative 3′ UTR of CRV15_07485 (*cmcH*), encoding 3′-hydroxymethylcephem-*O*-carbamoyltransferase, which is involved in the later steps of cephamycin C biosynthesis (Fig. 3; see Fig. S9B in the supplemental material) (42). The transcription of this long 916-bp *cmcH*-*ccaR* intergenic region was validated by RT-PCR, suggesting the presence of regulatory elements, such as an ARE box which is binding site of butyrolactone receptor protein Brp (41, 57). Additionally, this study identified the novel potential of a *cis*-regulatory RNA to bind the antisense strand of an alternative 3′ UTR. Taken together, the genome-wide determination of noncoding TUs could lead to the discovery of novel regulatory elements, including potential *cis*-regulatory RNAs involved in 3′-UTR regulation.

10.1128/mSystems.01013-20.9 FIG S9 Alternative 3′ UTRs determined by secondary TEPs isoforms and profile examples of premature transcription units. (A) Distribution of the number of TEP isoforms of the total 928 genes. (B) An example of potential *cis*-regulatory RNA binds to the antisense strand of the alternative 3′-UTR region. Red transparent box represents the long *cmcH*-*ccaR* intergenic region, and green transparent box represents the alternative 3′-UTR region of CRV15_07485 (*cmcH*). (C) An example of the premature TU profile with the largest standard deviation of the log_2_ RNA read-count ratio, i.e., the premature TU of CRV15_07890. (D) An example of the premature TU profile in the cephamycin C cluster, i.e., the premature TU of CRV15_07445. (E) An example of an antibiotic-resistance-associated premature TU profile, i.e., CRV15_17760. (F) Premature transcription unit profile of CRV15_14080 which may be related to antibiotic resistance. Download FIG S9, TIF file, 0.9 MB. Copyright © 2021 Hwang et al. 2021 Hwang et al. https://creativecommons.org/licenses/by/4.0/ This content is distributed under the terms of the Creative Commons Attribution 4.0 International license.

## DISCUSSION

In this study, we reported the following: (i) 1,427 TEPs with their unique structural and regulatory features; (ii) 1,648 TUs, including 150 premature TUs and 39 noncoding Tus; (iii) 610 TUCs, which are broad functional units; and (iv) the TU architecture of XRE-DUF397 pairs, which are the potential secondary metabolism regulators in *S. clavuligerus*. The TEPs determined herein included not only transcription termination sites but also enriched 3′-end termini after posttranscriptional processing. All TEPs were categorized into three classes in accordance with their bimodal distribution of folding free energy and the uridine counts downstream of the stem structure, namely, HS-U-rich, HS-U-lack, and LS-TEPs. The median value of HS-TEPs was much lower than previously reported 3′-end positions from term-seq of other species (14, 16, 18). Rather, the median value of LS-TEPs was comparable to them. However, the median value of folding free energy distribution of the random intergenic sequence in *S. clavuligerus* (−15 kcal/mol, same with LS-TEPs) was also very low compared with that of other species. Thus, the overall folding free energy distribution is very low in *S. clavuligerus*, probably due to the high GC content in the genome. Thus, the difference between HS-TEPs and LS-TEPs would be the presence of the RNA stem-loop structure. LS-TEPs did not have a significant stem-loop structure although LS-TEPs have much lower folding free energy compared with random sequences in other species. According to the previously reported features (31), HS-U-rich TEPs were mostly the intrinsic terminators, wherein highly structured GC stem loops, U-rich tracts, and A-tracts were enriched upstream from the GC stem-loop with a low read-through fraction. Second, HS-U-lack TEPs may involve I-shaped intrinsic terminators of the GC-rich eubacterial genome (36) or 3′-end termini of Rho-dependent-terminated transcripts sharing similar characteristics to those in E. coli (14). Third, LS-TEPs without a stem-loop structure were speculated as Rho-dependent termination sites owing to the lack of distinct sequence characteristics sharing diffused features of Rho-dependent terminators of E. coli (33) or termination sites by other mechanisms. For example, the folding free energy distribution of TEPs of archaea Sulfolobus acidocaldarius was almost identical to the random sequences, but the RNA-seq read density was significantly decrease at the TEPs, suggesting the potential presence of strain-specific transcription termination mechanisms (15). Otherwise, LS-TEPs could be the posttranscriptional processing sites by other mechanisms, including RNA binding proteins or endonucleases. Although many regulatory elements might be mixed in each TEP class, they have distinct sequence features, such as bimodal distribution of folding free energy, leading distinct potential biological roles.

Genome-wide TUCs were also determined in this study. For TUCs containing multiple genes, the expression level of each gene would be fine-tuned by regulating the transcript abundance for TU isoforms. Transcription of TU isoforms is generally regulated by alternative TSSs during transcription initiation (18). Here, NT-TEP analysis of TUCs suggested that alternative TEPs could also serve as regulatory elements for TU isoforms during transcription termination and posttranscriptional processing. In *Streptomyces* smBGCs, multiple serial reactions and complex regulatory networks must be tightly regulated to rapidly respond to dynamic environmental changes. The general mechanism involved in regulating transcription initiation is less efficient than the one which regulates transcript abundance by transcription termination and posttranscriptional processing, for rapid changes in protein expression (58). Potential explanations for these phenomena are supercoiling or relieving of RNA stem structures at the TEP by nucleosome-associated proteins and other regulatory proteins or RNA-RNA hybridization by noncoding RNA and the change in the protein abundance of termination factors, including Rho or processing factors, including RNases (37).

A significant finding was the abundance of TUs, including XRE-DUF397 pairs in *S. clavuligerus*. These gene pairs constituted the largest portion of TUC functions and had specific single nucleotide polymorphism (SNP) variations and specific targets. Moreover, XRE and DUF397 seemed to have coevolved within the same pair, and their protein abundance ratio differed among different pairs, although most pairs displayed a markedly higher abundance of DUF397 rather than the XRE. Most XRE-DUF397 TUs displayed bidirectional TEP at their 3′-end termini, which is the regulatory element for efficient termination of transcripts at both ends of the opposite strands via transcriptional interference. Some of the opposite gene pairs of XRE-DUF397 with Bi-TEPs were associated with resistance of toxic intermediates from secondary metabolism. XRE-DUF397 is potentially associated with secondary metabolism with tightly regulated transcriptional termination.

Genome-wide determination of TUs also revealed noncoding TUs, including 96 novel premature TUs and 27 novel regulatory RNAs. Potential novel riboregulators and riboswitches, which had not been predicted by computational approaches, were found. For example, the premature TU of CRV15_07890, which encodes an NADP-dependent malic enzyme, might be a specific metabolite-responsive riboregulator (Fig. S9C). This enzyme governs the anaplerotic reaction converting pyruvate to malate (a major tricarboxylic acid [TCA] cycle intermediate), and its concentration is usually decreased at the stationary phase due to its consumption for secondary metabolism (59). The novel premature TUs were related to three potential antibiotic-responsive riboregulators. CRV15_07445 (*pbpA*) is involved in resistance against β-lactam antibiotics in cephamycin smBGCs (42); thus, the premature TU of the gene may be a novel riboregulator that responds to cephamycin C or its intermediates (Fig. S9D). The premature TUs CRV15_17760 and CRV15_14080, which encode an aminoglycoside phosphotransferase and a penicillin-binding protein, respectively, may be additional novel riboregulators activated in response to other types of antibiotics (Fig. S9E and F). In conclusion, integrated analysis of term-seq data with multiomics data (29) revealed not only genome-wide TEPs, TUs, and TUCs but also underestimated potential transcriptional regulatory elements of transcriptional termination and posttranscriptional processing. These regulatory features in *S. clavuligerus* were also conserved in S. lividans (28), suggesting the unique general regulatory features of *Streptomyces* sp. However, several differences in TU, TUC, and XRE-DUF397 between two strains suggested the presence of strain-specific regulations, particularly for smBGCs. Further detailed studies on multilayer transcriptional regulatory elements in various *Streptomyces* spp. are required to establish a rational engineering design for silent smBGC activation.

## MATERIALS AND METHODS

### Strains and cell growth.

*S. clavuligerus* ATCC 27064 spores were inoculated to 50 ml R5− liquid complex medium [103 g/liter sucrose, 0.25 g/liter K_2_SO_4_, 10.12 g/liter MgCl_2_·6H_2_O, 10 g/liter glucose, 0.1 g/liter Casamino Acids, 5 g/liter yeast extract, 5.73 g/liter *N*-tris(hydroxymethyl)methyl-2-aminoethanesulfonic acid (pH 7.2), 0.08 mg/liter ZnCl_2_, 0.4 mg/liter FeCl_3_·6H_2_O, 0.02 mg/liter CuCl_2_·2H_2_O, 0.02 mg/liter MnCl_2_·4H_2_O, 0.02 mg/liter Na_2_B_4_O_7_·10H_2_O, and 0.02 mg/liter (NH_4_)_6_Mo_7_O_24_·4H_2_O] and 8-g glass beads (3- ± 0.3-mm diameter) in a 250-ml baffled flask and were grown at 30°C and 250 rpm. The grown mycelium was then diluted 100-fold and transferred to fresh R5− medium for the culture of biological duplicates. Cells were harvested at the early-exponential, transition-, late-exponential-, and stationary-growth phases (29).

### Term-seq library preparation.

The harvested cells were washed with 3-ml washing buffer composed of 20 mM Tris-HCl (pH 7.5), 140 mM NaCl, and 5 mM MgCl_2_. Then, the cells were resuspended in 500-μl lysis buffer composed of 0.3 M sodium acetate (pH 5.2), 10 mM EDTA, and 1% Triton X-100. The resuspended cells were frozen with liquid nitrogen and then lysed by grinding using a mortar and pestle. The powdered cells were thawed and separated by centrifugation, and the supernatant was collected as the lysate. RNA samples were purified from the lysate using phenol-chloroform-isoamyl alcohol (Sigma-Aldrich, St. Louis, MO, USA) and precipitated in ice-cold 100% ethanol. After DNase I treatment at 37°C for 1 h, RNA samples were purified using phenol-chloroform-isoamyl alcohol and precipitated in ice-cold 100% ethanol. Then, rRNAs were removed from the DNase I-treated RNA sample using the Ribo-Zero rRNA removal kit for bacteria (Epicentre, Madison, WI, USA) according to the manufacturer’s instructions. The rRNA-depleted RNA samples were purified by ethanol precipitation, and 450 ng of each sample was used for term-seq library construction. To amplify the 3′ end of the transcripts, 150 μM DNA adapter (5′-NNAGATCGGAAGAGCGTCGTGT-3′) was ligated to the 3′ end of the RNA sample. Next, 5-μl RNA sample, 1-μl 150 μM DNA adapter, 1 μl T4 RNA ligase I (10 U/μl; New England BioLabs [NEB], Ipswich, MA, USA), 1-μl 10× T4 RNA ligase I buffer, 10 mM ATP (Thermo Fisher Scientific, Waltham, MA, USA), 2 μl dimethyl sulfoxide (DMSO), and 9.5-μl 50% polyethylene glycol 6000 (PEG 8000; New England BioLabs) were mixed and incubated at 23°C for 2.5 h. Then, 2.2× AMPure XP beads (Beckman Coulter, Brea, CA, USA) were added to the solution and purified twice to select the proper size of libraries with a ligated adapter. For fragmentation, 1-μl 10× fragmentation buffer (Thermo Fisher Scientific) was added to 9-μl adapter-ligated RNA and incubated at 72°C for 90 s. The reaction was stopped by adding 1-μl Stop solution (Thermo Fisher Scientific), and 2.2× AMPure XP beads were added to the solution and purified twice to select the proper size of fragmented libraries. From the fragmented RNA sequences ligated with DNA adapters, first-strand cDNA synthesis was performed by reverse transcription using Superscript III reverse transcriptase (200 U/μl; Thermo Fisher Scientific) with 10 μM reverse transcription primer, followed by the addition of 2.2× AMPure XP beads and the collection of synthesized DNA. Second-strand cDNA was synthesized by the ligation of the second adapter to the 3′ end of the sequence, followed by PCR amplification. Next, 150 μM second-strand DNA adapter was added to 5 μl of the cDNA sample with 1-μl T4 RNA ligase I (10 U/μl), 1-μl 10× T4 RNA ligase I buffer, 10 mM ATP, 2 μl DMSO, and 9.5-μl 50% PEG 8000 and incubated at 23°C for 8 h. Then, 1.8× AMPure XP beads were added to the solution and purified twice to select the proper size of libraries with a second ligated adapter. Double-stranded cDNA was amplified using Phusion high-fidelity DNA polymerase (Thermo Fisher Scientific) with indexed primers. Amplification was monitored using a CFX96 real-time PCR detection system (Bio-Rad Laboratories, Hercules, CA, USA) and stopped at the beginning of the saturation point (14 cycles). The amplified sample was purified by adding 0.8× AMPure XP beads to obtain a final volume of 12 μl for the library.

### High-throughput sequencing.

All constructed libraries were sequenced using the HiSeq 2500 rapid mode platform with 50-bp single-end reads (Illumina, San Diego, CA, USA). The sequencing results were demultiplexed and processed using the CLC Genomics Workbench (CLC Bio, Aarhus, Denmark). A total of 10,019,122 and 9,107,580 raw reads were generated for each replicate and trimmed based on their quality (quality score, 0.05; maximum ambiguous nucleotides, 2), adapter sequences (Action: Remove adapter, mismatch cost: 2, gap cost: 3, internal match minimum score: 9, end match minimum score: 9, trim bases: 2 bp of both ends) and length (>15 bp). The trimming steps yielded 98.18% and 90.63% of the raw reads. A total of 7,956,235 (80.88% of raw reads) and 5,461,893 (66.17% of raw reads) reads with an average read length of 47 bp were uniquely mapped to the completed genome (mismatch cost: 2, insertion cost: 3, deletion cost: 3, length fraction: 0.9, similarity cost: 0.9, and ignore nonspecific match), corresponding to 43- and 30-fold coverage, respectively. The mapped information was exported as BAM files, and the 5′ end of the mapped reads at each genomic position were counted as TEP peak raw counts.

### Determination of TEPs.

TEPs were determined using custom perl and python scripts and manually curated using a combined method from previous studies (16, 17). Based on the term-seq peak raw counts, TEPs were selected using a machine-learning algorithm with positive- and negative-control sets as inputs. For the positive-control learning set, the peak with maximum intensity among the peaks of a subcluster was selected to identify true-positive peaks among peak shadows. First, peaks with a distance of less than 100 bp were clustered. Then, adjacent peaks with a standard deviation of <25 from their position in the same cluster were additionally subclustered. Low-intensity peaks with less than four counts were discarded to select enriched peaks. Then, peaks that were not present in both biological replicates were removed. Approximately 3,500 peaks were selected, and additional criteria for peak height enrichment were applied. Because the measurement of deviation from the distribution of peak values indicates how large the value is compared to adjacent peak shadows, the Z-score was calculated as previously described (17) under strict criteria; peaks with a Z-score of >6 were collected, and a total of 124 peaks showing decreased RNA profiles near TEPs were manually selected. A total of 1,345 negative-control peaks were also manually selected from the −10- to +10-bp positions relative to the positive-control peak positions. TEPs were then searched using an in-house python script based on the scikit-learn package. Briefly, the number of 3′ ends from the high-throughput sequencing reads mapped from the −10- to +10-bp genomic positions were submitted to a machine classifier as a data set with a machine classifier call for +1 position as its output (that is, to determine TEP, the neighboring 20-bp sequencing profile was considered). Two different machine classifiers, namely, K-nearest neighbor (KNN) and support vector machine, were trained by the training set and showed a mean accuracy of 98.77% and 94.68% of upon cross-validation (trained with a random half of the training set; the performance on remaining 50% was measured, iterated 1,000 times), respectively. Thus, we used KNN for the further discovery of TEPs. The python script and KNN machine classifiers used (pickled python objects) are available online at http://cholab.or.kr. The final set of TEPs was selected from union sets of the replicates with manual curation.

### Determination of TUs and TUCs.

The TUs of *S. clavuligerus* were defined by integrating the information about transcription start sites (TSSs) from differential RNA sequencing (dRNA-seq), TEPs from term-seq, and RNA read count from RNA-seq (29). First, TSSs and TEPs were classified into two groups, namely, coding TUs and noncoding TUs. Then, all possible combinations of TSSs and TEPs were tested for their association by calculating the normalized average RNA read count sum between the 100-bp downstream region of the TSS and the 100-bp upstream region of the TEP. If the distance between the TSS and TEP was shorter than 200 bp, the normalized average RNA read count between the TSS and TEP without the end criteria was calculated. Then, the normalized average RNA read count of the 200-bp window region within the entire connected region was calculated and repeated for each +1-bp shifted window for 200 bp upstream of the TEP. The connected region between the TSS and TEP was classified as a TU when the average RNA read count of all windows was higher than 5% of the average RNA read count of the entire connected region for at least one growth phase. If the average RNA raw read count of the window was smaller than 5, it was excluded. Consequently, a total of 1,648 TUs were determined, and they were classified into 4 categories based on the number of genes within the TU and their location relative to the genes. For noncoding TUs, the genome sequences were scanned by the Infernal Rfam cmscan module to predict their function (50). The ribosome-protected mRNA fragment (RPF) value was calculated to predict novel small proteins among the noncoding TUs using ribosome profiling data described previously (29). TUCs were defined as a group of connected TUs overlapping the TU region within at least 1 bp. All TUs were assigned as nonterminal or terminal based on their TEP location relative to the longest TU of their TUC.

### RNA structure and motif analysis.

The TEP motifs were searched using MEME (zoops, *P < *0.05) among the extracted sequences, from 40 bp upstream to 20 bp downstream of the TEPs (60). The full extracted sequences with the motif were used as inputs for WebLogo 3 (61). The fold free energy of the TEPs was calculated using RNAfold software (62) using the 40-bp upstream sequences of the TEPs. The interaction frequency between 2 bases in the 100-bp upstream sequences was calculated from the RNAfold results as the ratio of the number of TEPs with the corresponding base interaction to the total number of TEPs. Nucleotide enrichment at each position was calculated as the fold change of base frequency relative to the average base frequency at 10,000 randomly chosen positions.

### RNA density, read-through fraction, and RNA read count analysis.

RNA read counts for each position were calculated based on the previous RNA-seq data of *S. clavuligerus* at all four growth phases, namely, early exponential, transition, late exponential, and stationary (29). For RNA read density, the max RNA read count between the −300 and +300 positions from TEP was normalized to 1, and RNA read counts at other positions were normalized relative to the max position. Then, all RNA read counts for TEPs at each position were added and divided by the sum of all normalized RNA read counts at −300 to +300. The four normalized RNA read counts from the four growth phases were averaged. To determine the read-through fraction, the sum of normalized RNA counts at 0 to +300 was divided by the sum of normalized RNA read counts at −300 to 0 from the TEP, and the 4 read-through fractions from the 4 growth phases were averaged. To calculate the RNA ratio of coding DNA sequence (CDS) to premature TU and the RNA expression levels, the reads per kilobase per million mapped reads (RPKM) value was used.

### Functional enrichment analysis.

The functional enrichment of Clusters of Orthologous Groups (COG) functional categories of the genes were calculated as the ratio of the gene number for one COG category to the number of total COG categories. Genes without any assigned COG category were not counted. For example, if 4 genes within a TUC were classified into the COG categories L, L, C, and not assigned, the functional enrichment score would be 2/3. The enrichment of antiSMASH functional categories for the smBGC genes was calculated using the same method used for COG functional categories.

### Comparative analysis of the amino acid sequence of XRE and DUF397.

The protein sequences of XRE and DUF397 in XRE-DUF397 pairs were aligned, and their pairwise distances were calculated using MEGA X (63). Sequence alignment was performed using the ClustalW algorithm with the BLOSUM protein weight matrix, and all other parameters for the alignment were set to default values. The pairwise distance between two proteins was calculated using the Poisson model with no variance estimation method. The sequence alignment figure containing the consensus sequence, identity, and similarity information at each position was exported from Geneious 11.1.2 with 13 bases/residues at 100% zoom (64).

### Data availability.

The full-genome sequences and annotations can be found at the National Center for Biotechnology Information as GenBank accession numbers CP027858 and CP027859. All raw sequencing data of RNA-seq, dRNA-seq, and ribosome profiling can be found in the GEO under the accession number GSE128216. Raw sequencing data of term-seq can be found in the GEO under the accession number GSE138325.
