# Supplementary figures and images for: Elucidating the Regulatory Elements for Transcription Termination and Posttranscriptional Processing in the Streptomyces clavuligerus Genome
Source: mSystems. 2021 May 4;6(3):e01013-20. doi: 10.1128/mSystems.01013-20 (PMC8269248; doi:10.1128/mSystems.01013-20)

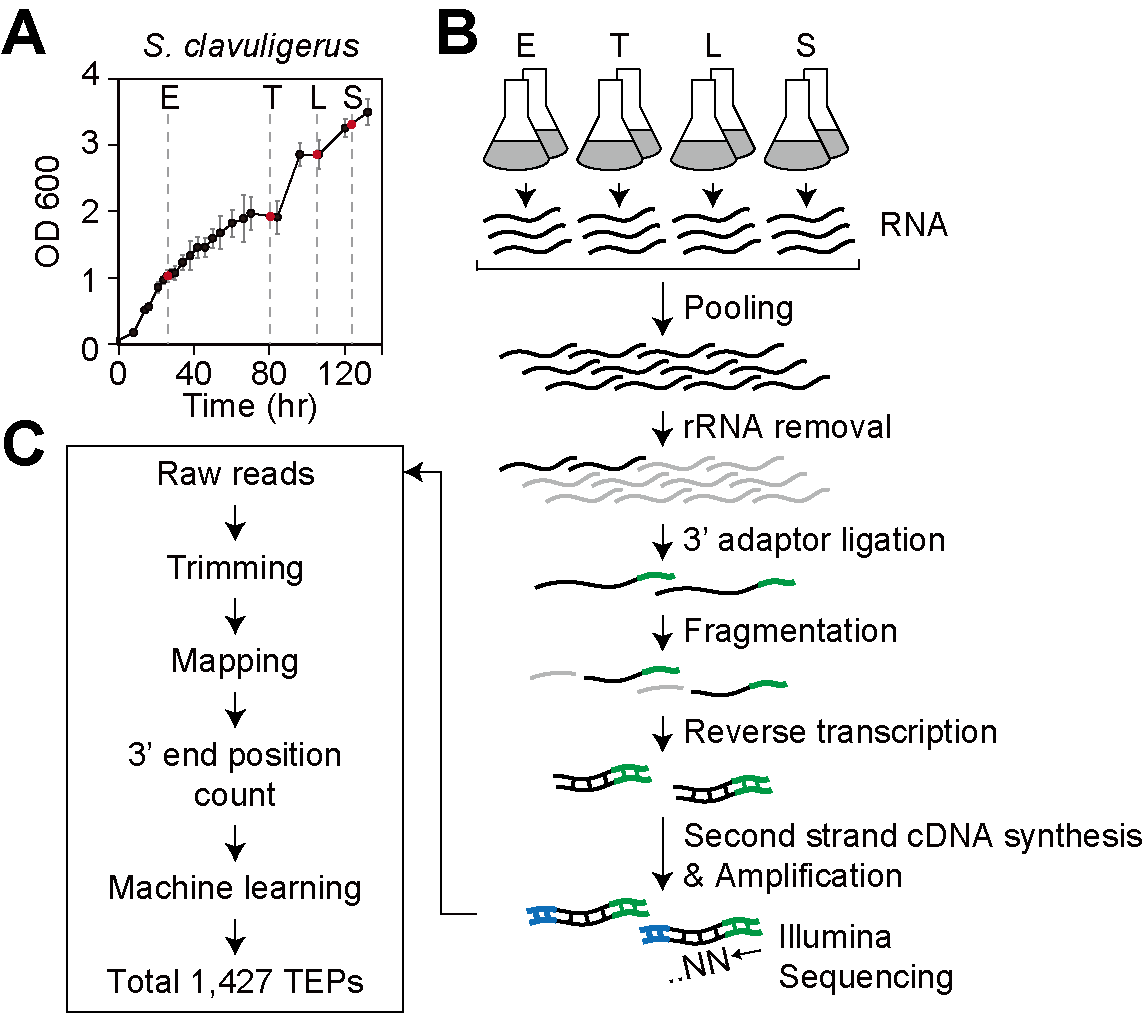

Supplement: FIG S1 [file msystems.01013-20-sf001.tif]

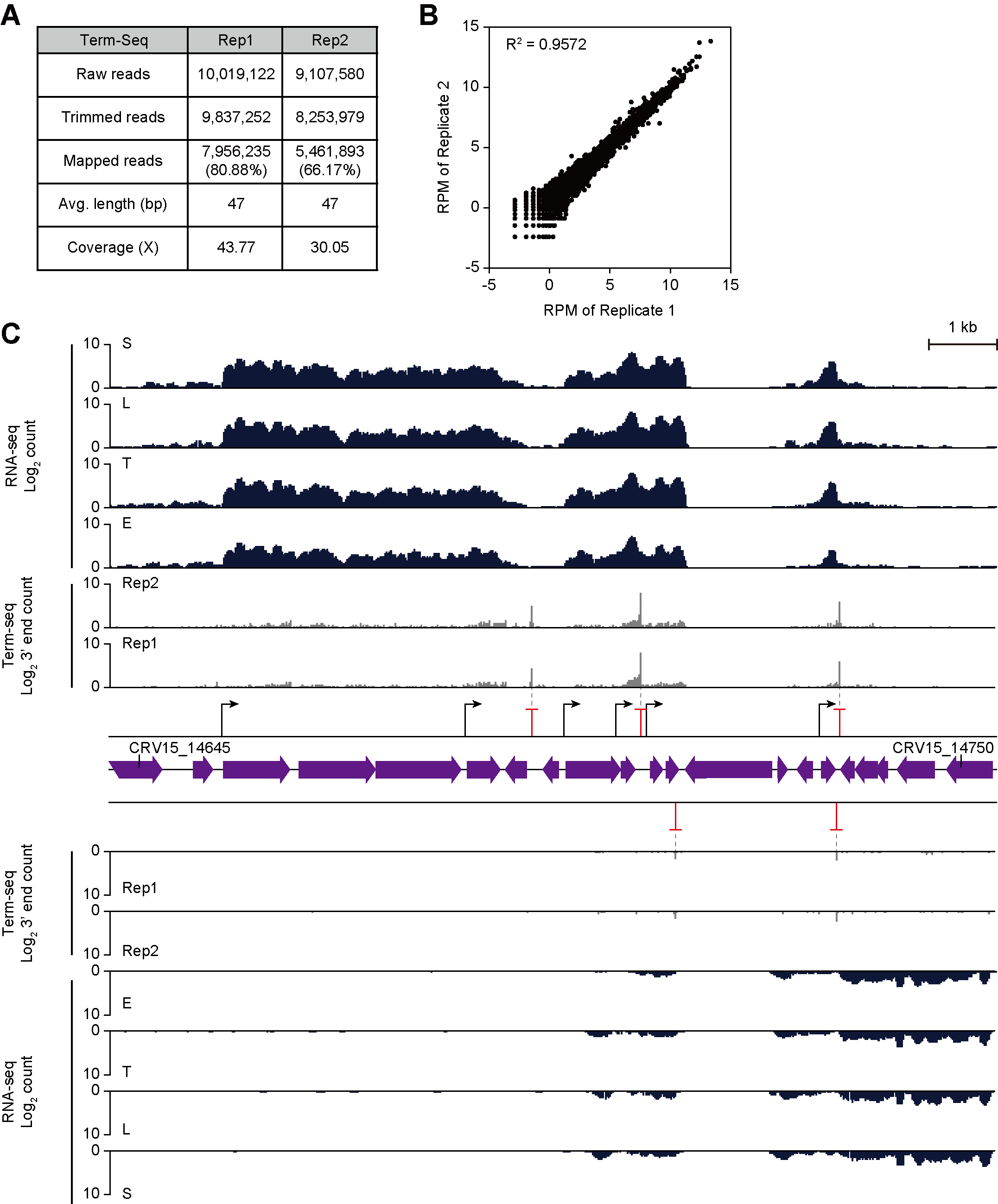

Supplement: FIG S2 [file msystems.01013-20-sf002.tif]

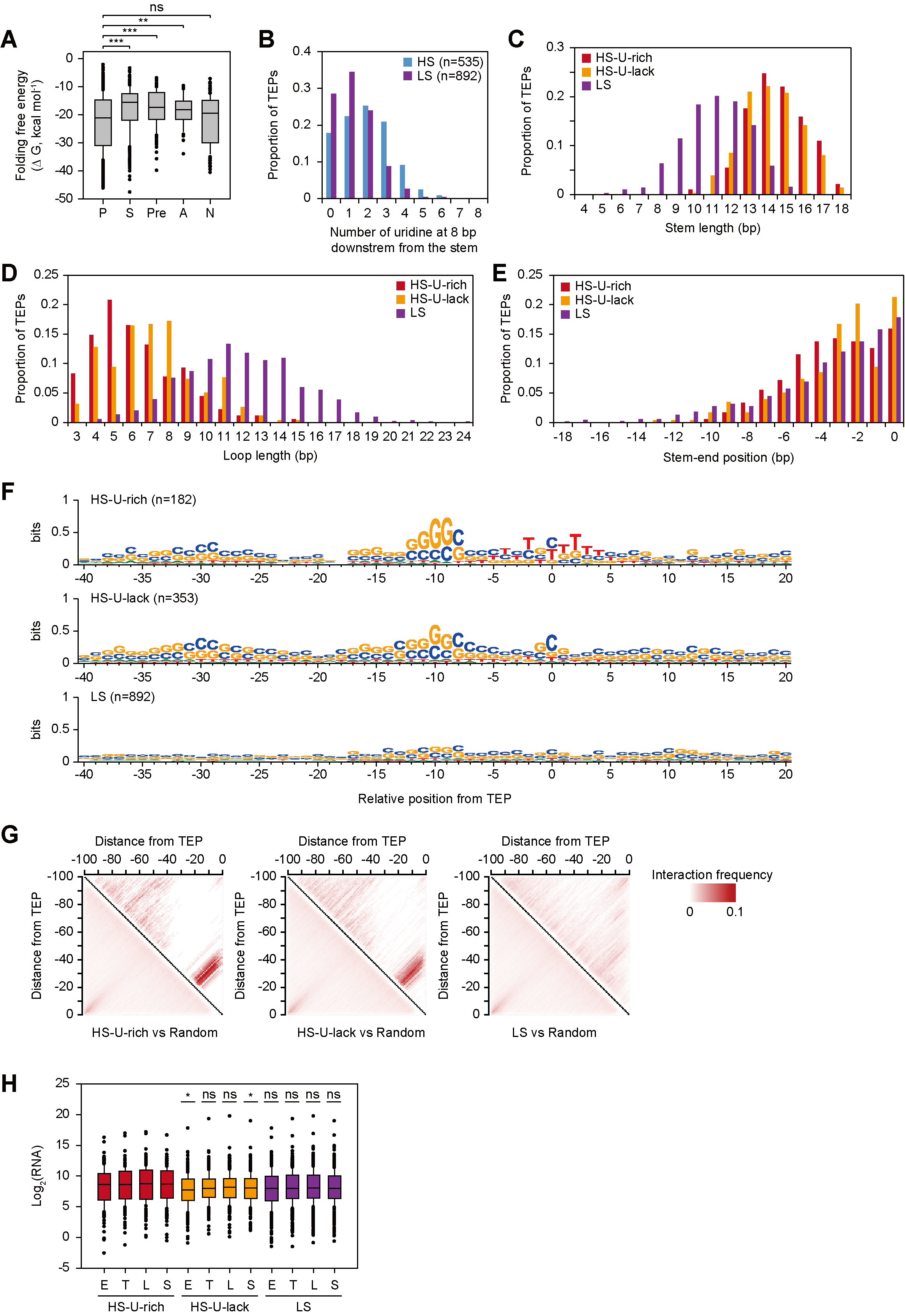

Supplement: FIG S3 [file msystems.01013-20-sf003.tif]

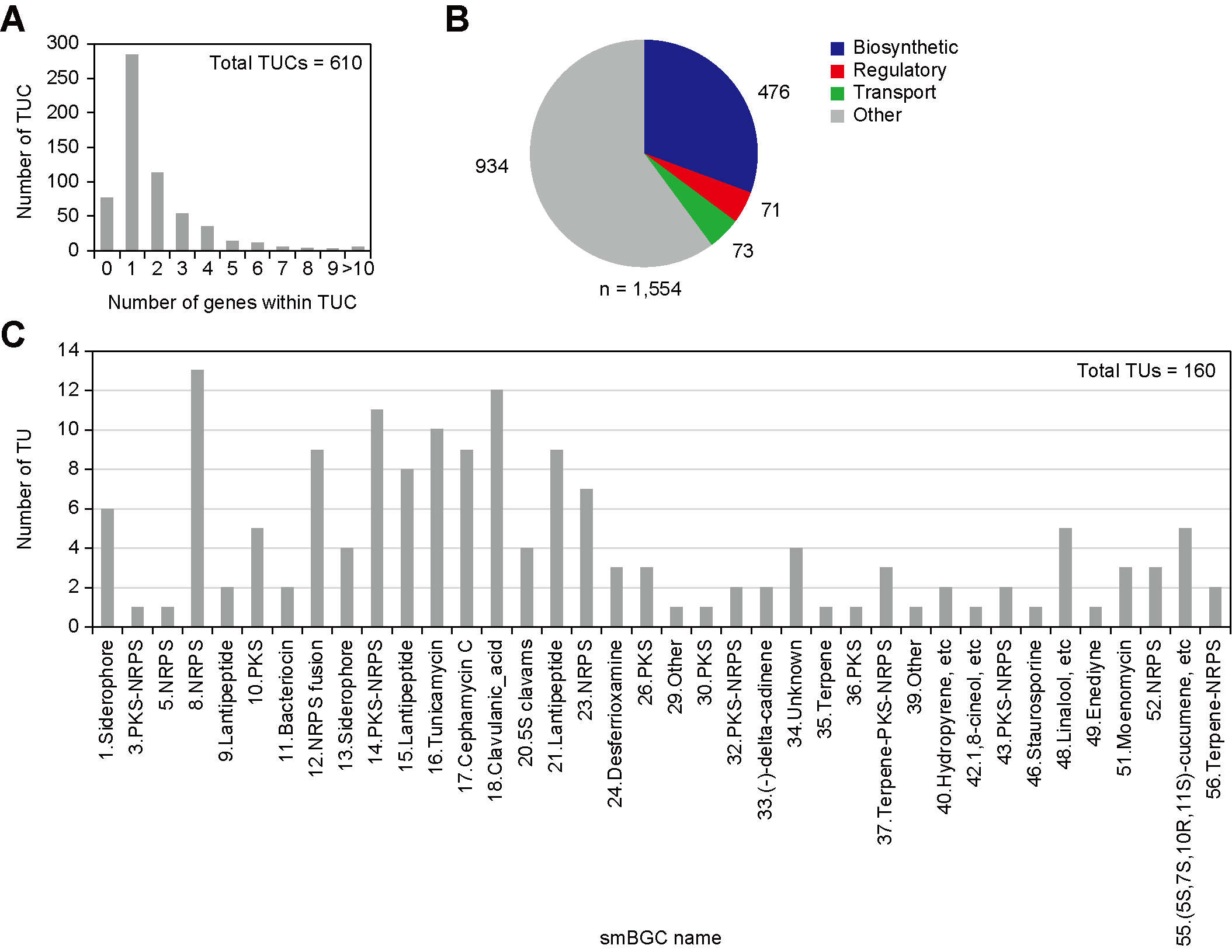

Supplement: FIG S4 [file msystems.01013-20-sf004.tif]

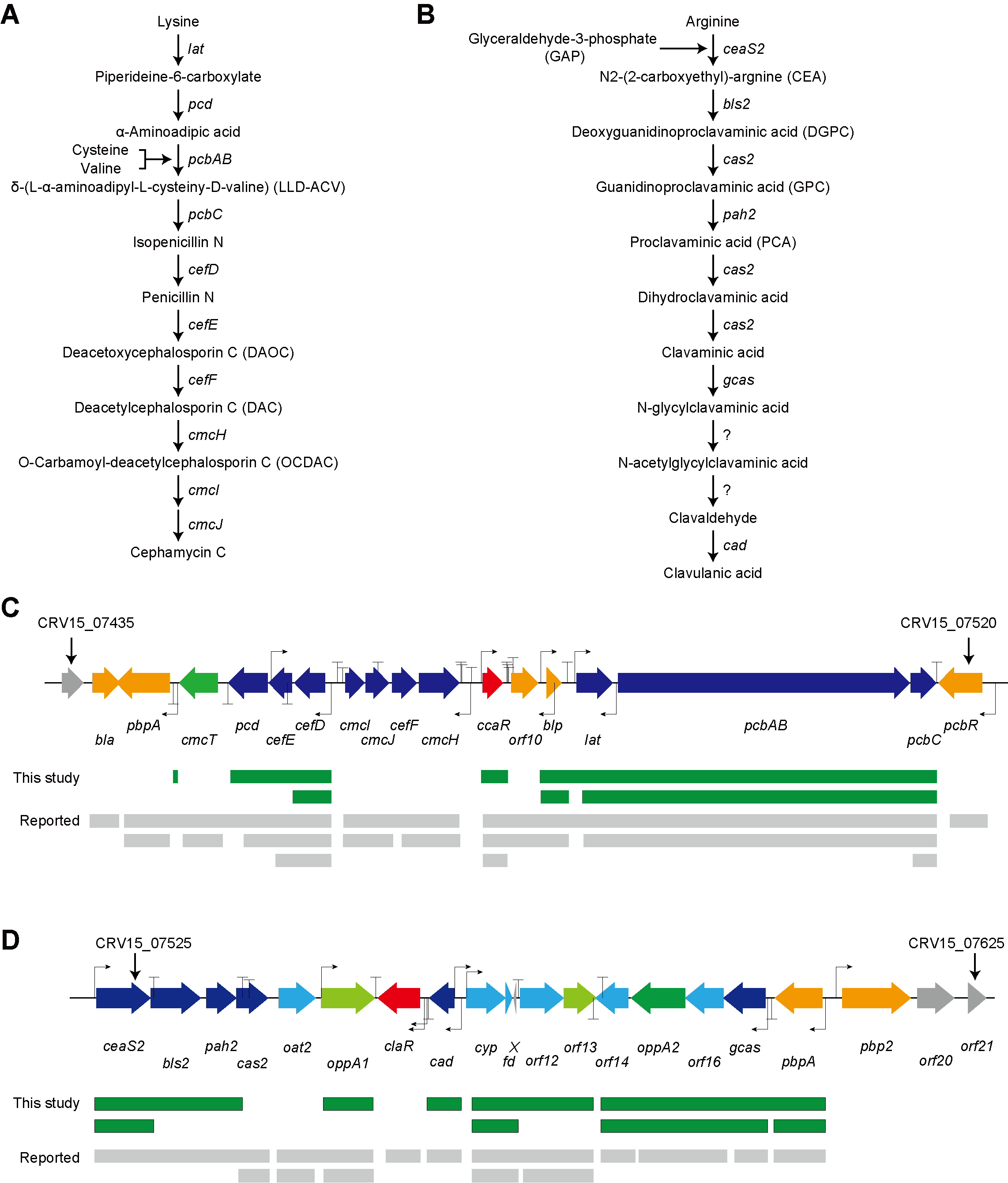

Supplement: FIG S5 [file msystems.01013-20-sf005.tif]

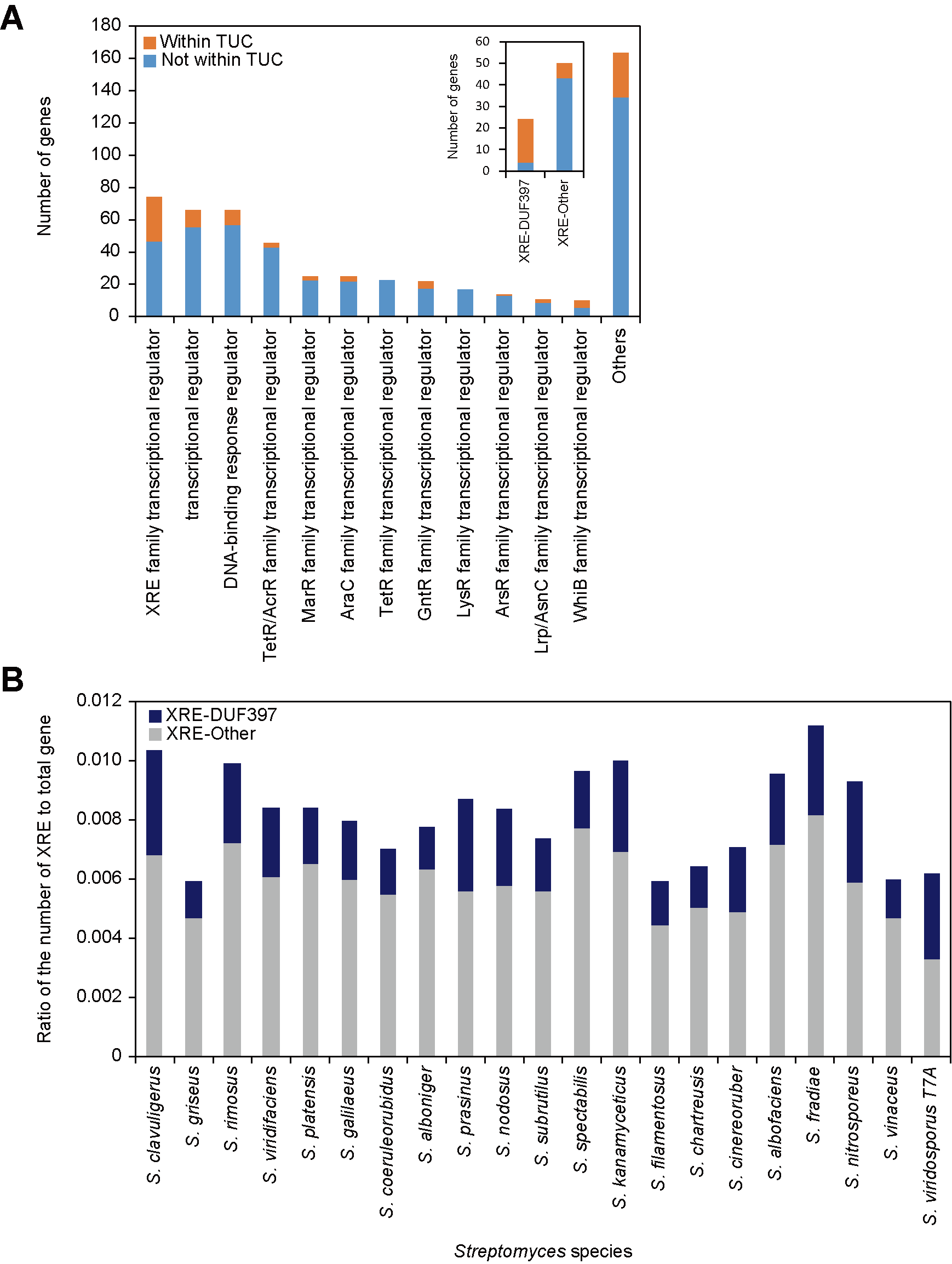

Supplement: FIG S6 [file msystems.01013-20-sf006.tif]

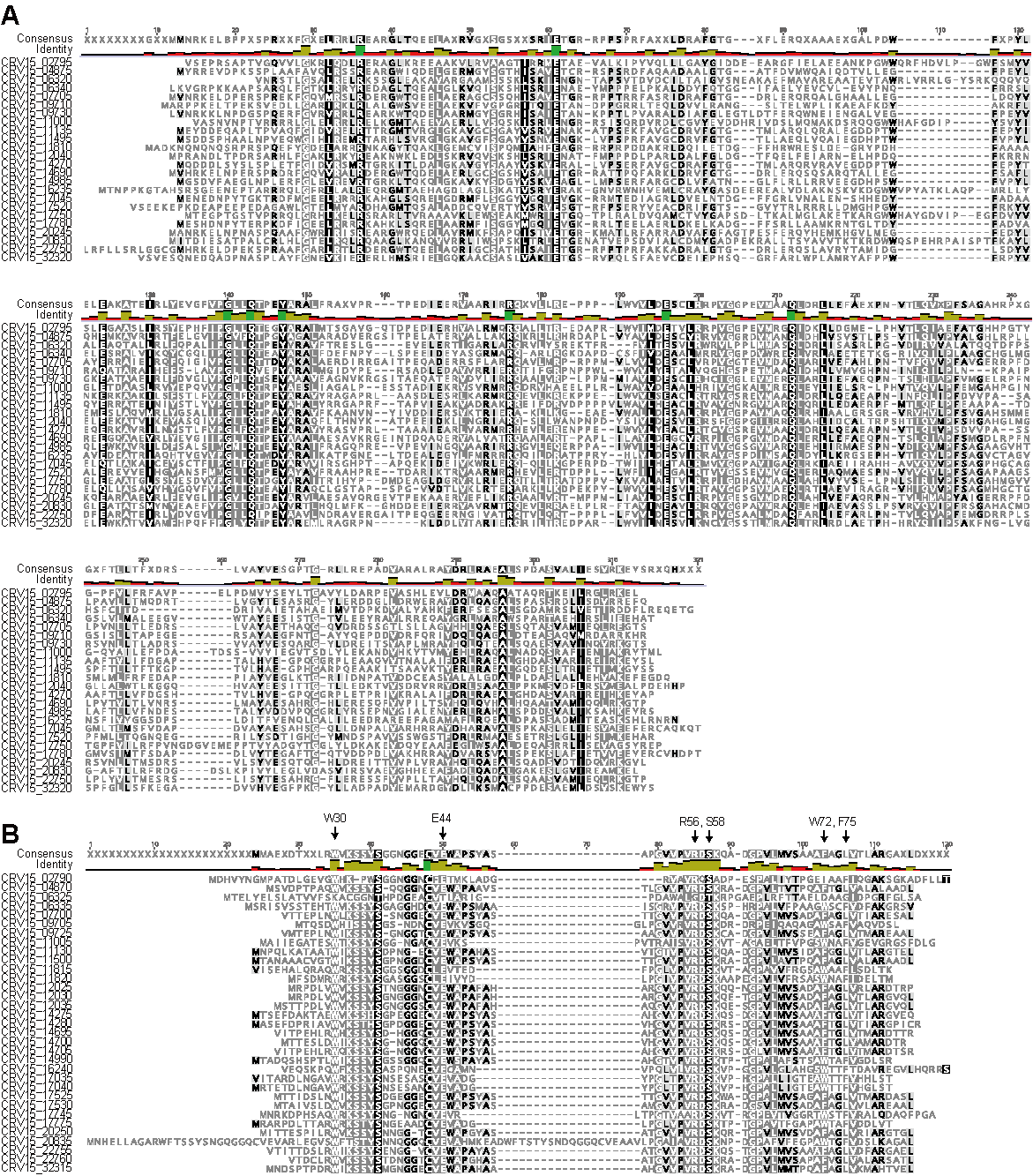

Supplement: FIG S7 [file msystems.01013-20-sf007.tif]

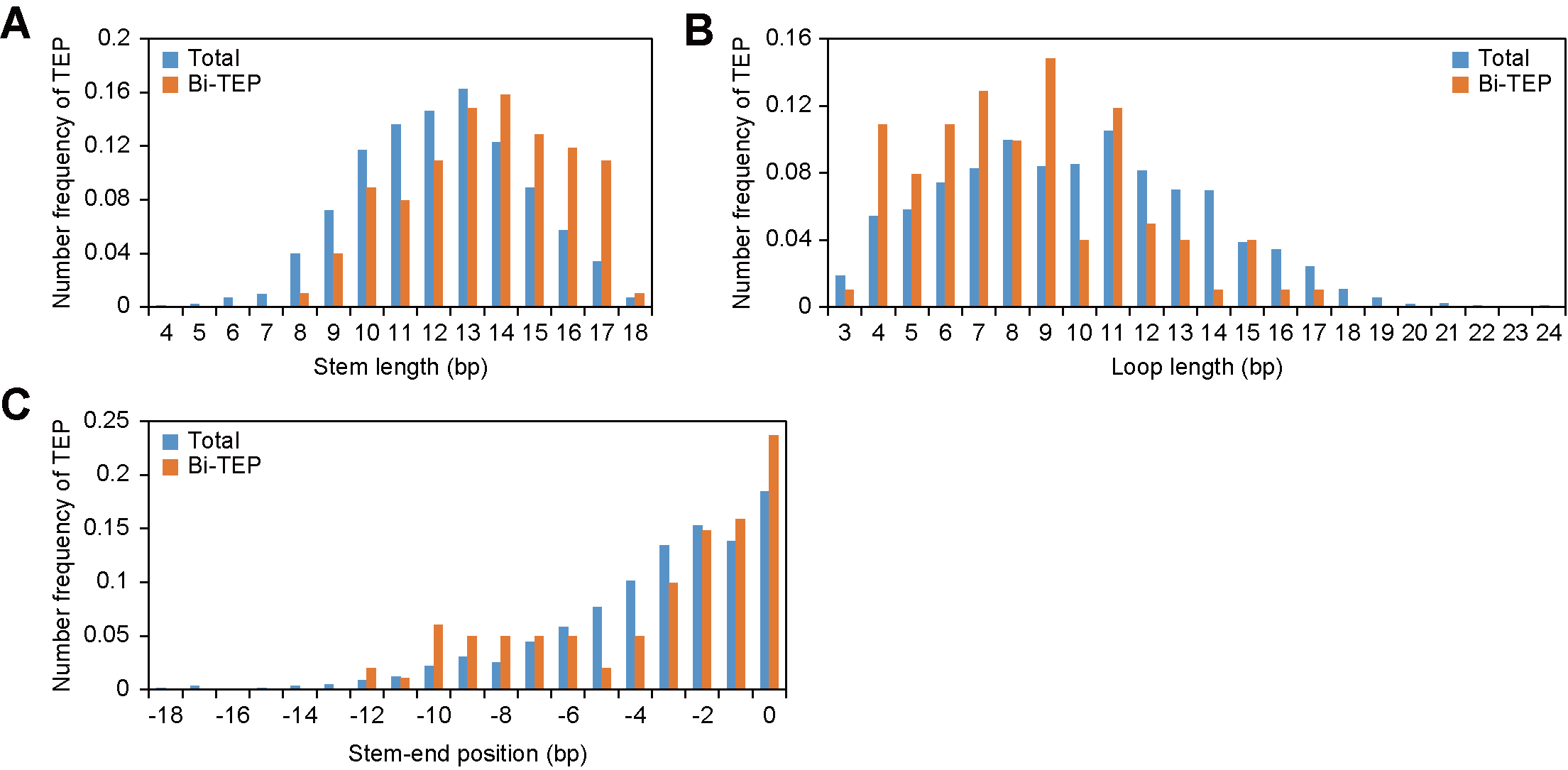

Supplement: FIG S8 [file msystems.01013-20-sf008.tif]

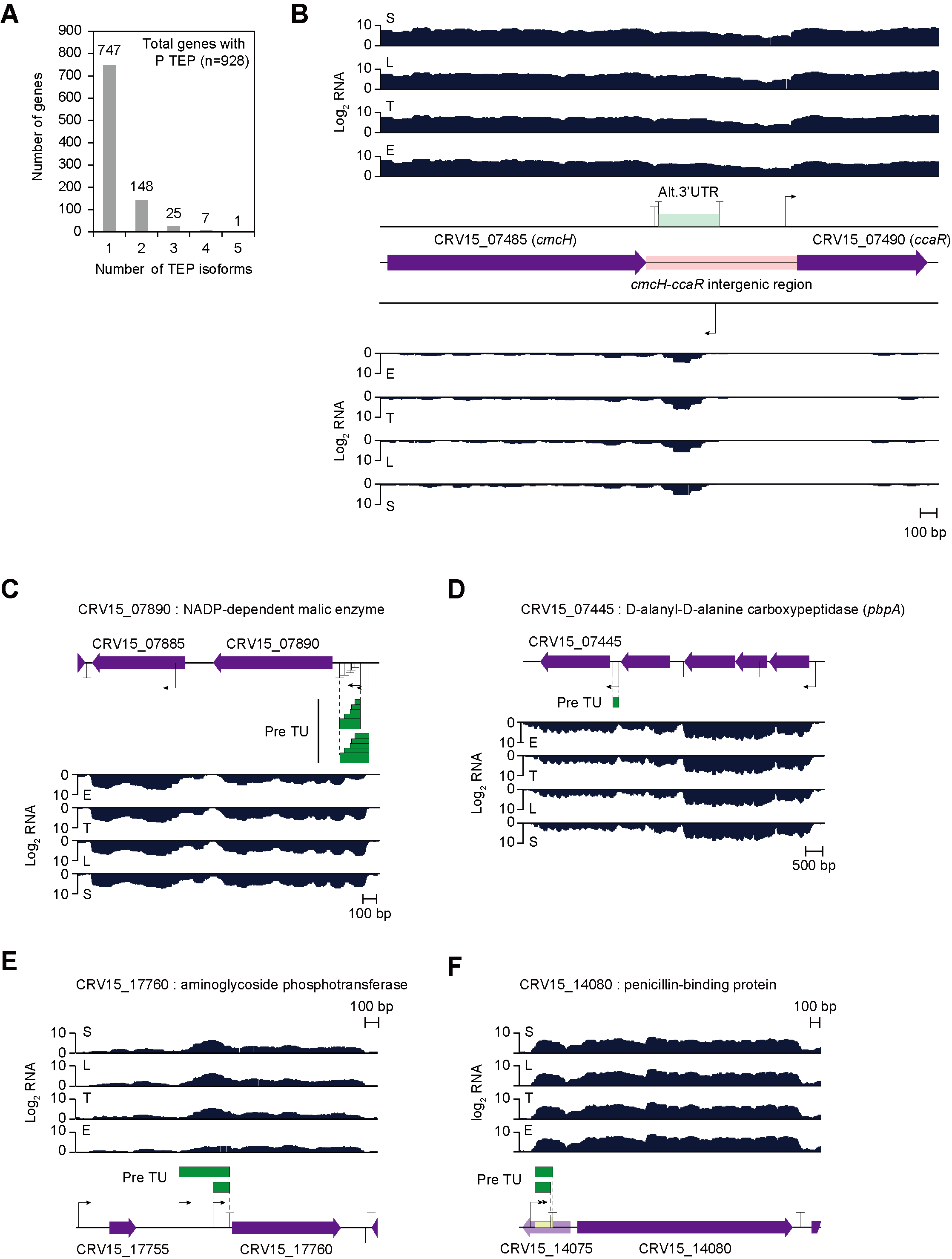

Supplement: FIG S9 [file msystems.01013-20-sf009.tif]
